# Growth and Photosynthetic Activity of Selected Spelt Varieties (*Triticum aestivum* ssp. *spelta* L.) Cultivated under Drought Conditions with Different Endophytic Core Microbiomes

**DOI:** 10.3390/ijms21217987

**Published:** 2020-10-27

**Authors:** Karolina Ratajczak, Hanna Sulewska, Lidia Błaszczyk, Aneta Basińska-Barczak, Katarzyna Mikołajczak, Sylwia Salamon, Grażyna Szymańska, Leszek Dryjański

**Affiliations:** 1 Department of Agronomy, Poznan University of Life Sciences, 11 Dojazd St., 60-632 Poznań, Poland; sulewska@up.poznan.pl (H.S.); grazyna.szymanska@up.poznan.pl (G.S.); 2 Institute of Plant Genetics, Polish Academy of Sciences, 34 Strzeszyńska St., 60-479 Poznań, Poland; lgol@igr.poznan.pl (L.B.); abas@igr.poznan.pl (A.B.-B.); kmiko@igr.poznan.pl (K.M.); ssal@igr.poznan.pl (S.S.); 3 Agrii Poland Ltd., 233 Obornicka Str., 60-650 Poznań, Poland; leszek.dryjanski@agrii.pl

**Keywords:** arbuscular mycorrhizal (AM), abiotic stress, fungal diversity, chlorophyll fluorescence, photosynthesis

## Abstract

The role of the microbiome in the root zone is critically important for plants. However, the mechanism by which plants can adapt to environmental constraints, especially water deficit, has not been fully investigated to date, while the endophytic core microbiome of the roots of spelt (*Triticum aestivum* ssp. *spelta* L.) grown under drought conditions has received little attention. In this study, we hypothesize that differences in the endophytic core of spelt and common wheat root microbiomes can explain the variations in the growth and photosynthetic activity of those plants, especially under drought conditions. Our greenhouse experimental design was completely randomized in a 2 × 4 × 3 factorial scheme: two water regime levels (well-watered and drought), three spelt varieties (*T. aestivum* ssp. *spelta* L.: ‘Badenstern’, ‘Badenkrone’ and ‘Zollernspelz’ and one wheat variety: *T. aestivum* ssp. *vulgare* L: ‘Dakotana’) and three mycorrhizal levels (autoclaved soil inoculation with *Rhizophagus irregularis*, control (autoclaved soil) and natural inoculation (non-autoclaved soil—microorganisms from the field). During the imposed stress period, relative water content (RWC), leaf chlorophyll fluorescence, gas exchange and water use efficiency (WUE) were measured. Microscopic observations of the root surface through fungi isolation and identification were conducted. Our results indicate that ‘Badenstern’ was the most drought tolerant variety, followed by ‘Zollernspelz’ and ‘Badenkrone,’ while the common wheat variety ‘Dakotana’ was the most drought sensitive. Inoculation of ‘Badenstern’ with the mycorrhizal fungi *R. irregularis* contributed to better growth performance as evidenced by increased whole plant and stalk dry matter accumulation, as well as greater root length and volume. Inoculation of ‘Zollernspelz’ with arbuscular mycorrhizal fungi (AMF) enhanced the photochemical efficiency of Photosystem II and significantly improved root growth under drought conditions, which was confirmed by enhanced aboveground biomass, root dry weight and length. This study provides evidence that AMF have the potential to be beneficial for plant growth and dry matter accumulation in spelt varieties grown under drought conditions.

## 1. Introduction

Spelt (*Triticum aestivum* ssp. *spelta* L.) is an “ancient” crop that is increasingly used in the food industry due to the trend of introducing non-common wheat cereals to derive bakery products (so-called “superfoods”) [1] with multiple functional benefits [2] that contain elevated amounts of particular nutrients. Since the late 1990s, interest in spelt cultivation has increased, and the cultivation area of this ancient species is likely to increase significantly in the future. Concomitant with this potential increase in cultivation area is reduced pesticide use, which may be difficult to achieve in practice, especially as monoculture cropping increases the pressure from disease and pests. Common wheat (*T. aestivum* ssp. *vulgare* L.) is a major, modern crop that accounts for about 95% of the 700 million tons of wheat that is grown annually worldwide in monoculture but is very susceptible to disease, with yield losses estimated to be as great as 90% under high pressure from fungal diseases, without fungicide protection [3]. In contrast to common wheat cultivated in high input intensive systems [4], spelt is usually grown in organic or traditional low input farming on a small scale [5]. The morphological structure of the spelt kernel (hard leathery chaff) protects the kernel from air pollution, and makes spelt less susceptible to pests, in contrast to common wheat, and is suitable for organic farming, where the use of synthetic pesticides and fertilizers is strictly forbidden [6]. Moreover, it complies with EU rules for integrated plant cultivation and the reduction of the negative impact of intensive agriculture on the environment [7].

Current climate scenarios indicate a severe impact on food production in the future [8]. The intensity and magnitude of combined high-temperatures and drought stress are projected to further intensify under increasing atmospheric carbon dioxide (CO_2_) concentrations. The protection of crop plants from the effects of global climate change, which may negatively affect yields, is difficult, or even impossible, to implement. However, our knowledge of microbiota is constantly expanding due to the application of different cultivation-independent techniques based on the isolation of all 16S rRNA present in plants, which are, consequently, dedicated to the detection of both cultivable and non-cultivable endophytes [9]. New techniques have significantly improved the understanding of the role of the plant microbiome and have proven that a plant can adapt to a changing environment by involving different components of its microbiome through the selection of at least part of their ecto- and endospheric microbiota to better adapt to environmental constraints [10]. Indeed, adaptation to local environmental conditions is valuable in relation to droughts, particularly for cereals, where many studies have shown that the impact of drought stress occurs at the agronomic, physiological and molecular levels [11]. Following recent research into plant microbiota, a new model was constructed for plant image, as a consequence of the plant per se and its unique microbiota, which collectively closely integrated with the host form a ‘plant holobiont’ [12]. The beneficial advantages that microbes provide for their host include the facilitation of nutrient absorption from the soil (e.g., via mycorrhizal formation) [13] that could mitigate the effects of drought, which limits shoot elongation, leaf area and tillering by decreased CO_2_ assimilation in the leaf, and slows nutrient mobilization to the growing tissues through decreased stomatal conductance, transpiration and low relative water content [14,15]. Water-limited conditions alter the time of flowering and, depending upon the duration and intensity of the drought stress, can even delay flowering [16], leading to pollen and spikelet termination at the early reproductive stage, thus decreasing grain numbers on the wheat stalk [17]. Moreover, the ability of the microbes to provide nitrogen for their host, especially in the later stages of development when severe drought can limit assimilation, could aid in maintaining a higher grain number and the 1000-grain weight [18]. In wheat plants, terminal drought at the grain filling stage results in early senescence with a shorter grain-filling duration, and low green flag-leaf area persistence [19]. Symbiotic microorganisms also provide protection against pathogens [20] and provide additional genes to the host, which extend the ability of the plant to adapt to many kinds of environmental conditions and changes [10]. Crop adaptive strategies to the environment (different stresses) are coordinated and fine-tuned by adjusting growth, development and cellular and molecular activities [21]. The upshot of better understanding such plant strategies is the substantial impact on a variety of research applications, for example, potential innovations in crop production, especially the improvement of plant growth in adverse conditions, such as drought.

Therefore, in this study, we hypothesize that differences in the endophytic core of spelt wheat varieties and common wheat root microbiomes can explain the variations in growth and photosynthetic activity, especially under drought conditions. Consequently, the aim of the research was also to identify the introduced microbiome, as well as the microbiome that naturally inhabits the root system in the field.

## 2. Results

### 2.1. Colonization of Roots by Fungi

**Figure 1:**
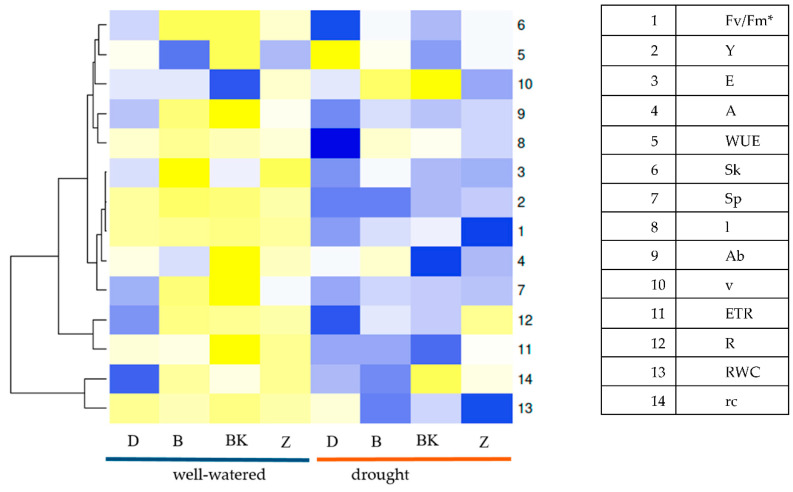
Comparable reaction between the physiological state of the plant, dry matter accumulation in the plant and root system in the common wheat variety ‘Dakotana’ (D), and spelt wheat varieties ‘Badenstern’ (B), ‘Badenkrone’ (BK) and ‘Zollernspelz’ (Z) under two water regimes (well-watered, drought). * Abbreviation as in Table 1: 1. Fv/Fm, 2. Y, 3. E., 4. A, 5. WUE, 6. Sk, 7. Sp, 8. l, 9. Ab, 10. v, 11. ETR, 12. R, 13. RWC, 14. rc. Scale represents high value (in yellow tint) to low value (in blue tint).

**Figure 2:**
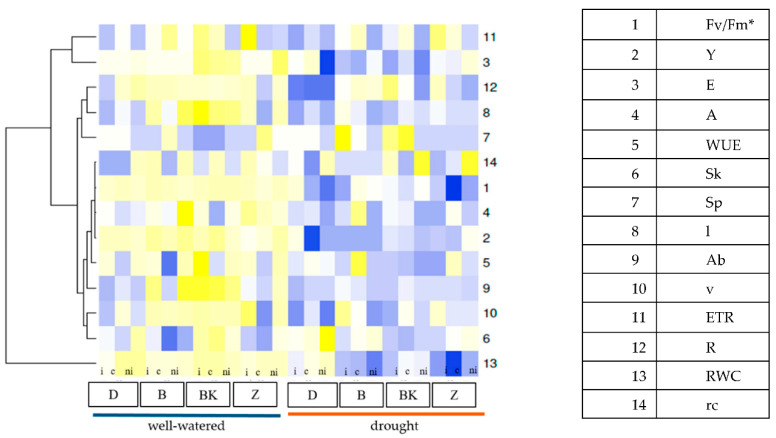
Comparable reaction between the physiological state of the plant, dry matter accumulation in the plant and root system in the common wheat variety ‘Dakotana’ (D), and spelt wheat varieties ‘Badenstern’ (B), ‘Badenkrone’ (BK) and ‘Zollernspelz’ (Z) in two water regimes (well-watered/drought) with different mycobiome composition in the roots (i—with *G. irregulare* inoculation, ni—natural inoculation in non-sterile soil, c—control, sterile soil). * Abbreviation as in Table 1: 1. Fv/Fm, 2. Y, 3. E., 4. A, 5. WUE, 6. Sk, 7. Sp, 8. l, 9. Ab, 10. v, 11. ETR, 12. R, 13. RWC, 14. rc. Scale represents high value (in yellow tint) to low value (in blue tint).

**Figure 3:**
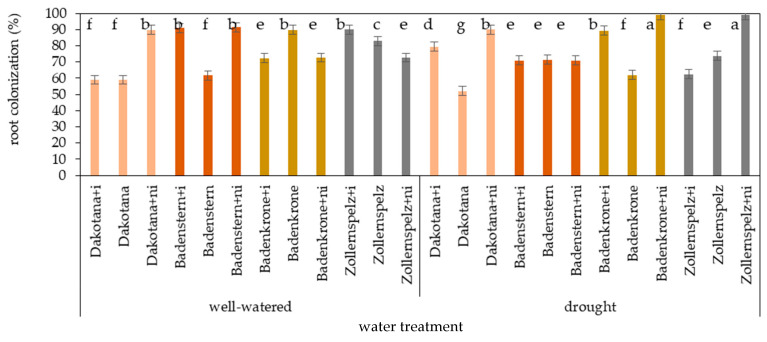
Root colonization by fungi (%) in wheat varieties with different mycobiome composition in roots (+i—with *G. irregulare* inoculation, +ni—natural inoculation) under well-watered and drought conditions. Variety without +i/ni—grown in sterile soil. Letters a–g indicate statistically different mean values at *p* < 0.05.

There were significant interactions between water regimes, varieties and mycobiome composition in the roots on root colonization (%) (*p* < 0.01) (Table 1, Figure 1 and Figure 2). Irrespective of mycorrhizal inoculation, average colonization of the roots in well-watered conditions was greatest in the spelt wheat varieties ‘Zollernspelz’ (81.9%) and ‘Badenstern’ (81.3%), and least in the common wheat variety ‘Dakotana’ (69.2%). Under drought conditions, root colonization of ‘Badenstern’ (71.0%) and ‘Dakotana’ (73.8%) was lowest, while the largest value was observed in ’Badenkrone’ (83.4%) (Figure 3). In well-watered conditions, mycorrhizal inoculation caused a significant increase in root colonization by fungi in ‘Badenstern’ (by 29 p.p.) and ‘Zollernspelz’ (by 7 p.p.), whereas a decrease (by 18 p.p.) was observed in ‘Badenkrone,’ in comparison to the sterile soil (control). Colonization of the roots in the common wheat variety ‘Dakotana’ was at the same level (59%) in the inoculated and non-inoculated plants. Mycorrhizal colonization of ‘Dakotana’ and ‘Badenkrone’ with *G. irregulare* increased root colonization by fungi, while root colonization in ‘Badenstern’ and ‘Zollernspelz’ decreased under drought stress. Under drought conditions, the greatest root colonization was observed in spelt wheat varieties ‘Badenkrone’ (99%) and ‘Zollernspelz’ (99%) with natural inoculation. Similarly, natural inoculation significantly increased symbiosis in the roots of ‘Dakotana’ by 30 and 38 p.p. in well-watered and drought conditions, respectively. Additionally, natural inoculation increased the colonization of fungi in the roots of ‘Badenkrone’ and ‘Zollernspelz’ (Figure 2 and Figure 3). 

### 2.2. Physiological State of Plants during the Stress Period Induced in the BBCH 65–69 Phase

During water stress, irrespective of microorganism inoculation, the common wheat variety ‘Dakotana’ exhibited higher average Fv/Fm, A, Y, E, RWC and WUE values than the spelt varieties. Variety ‘Badenstern’ appeared to have the photosynthetic apparatus best prepared to deal with drought stress evidenced by greater photosynthetic and transpiration rates in comparison to the other varieties (Figure 1). Regardless of variety, plants with AM inoculation under drought conditions showed the greatest ETR and E values, plants with natural inoculation showed greater Fv/Fm, Y and RWC values, while plants grown in the sterile soil (control) exhibited greater ETR, E and A values than the plants grown in the inoculated soils.

**Figure 4:**
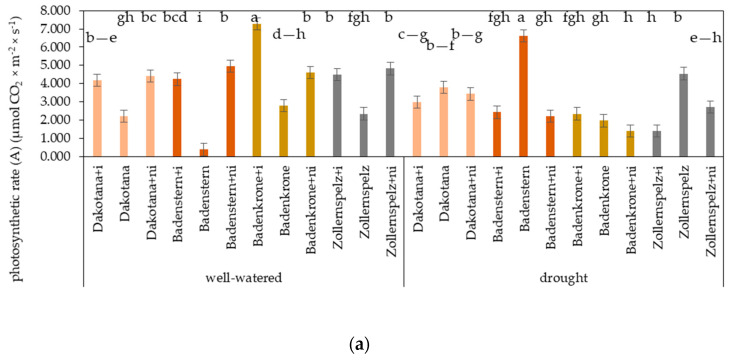

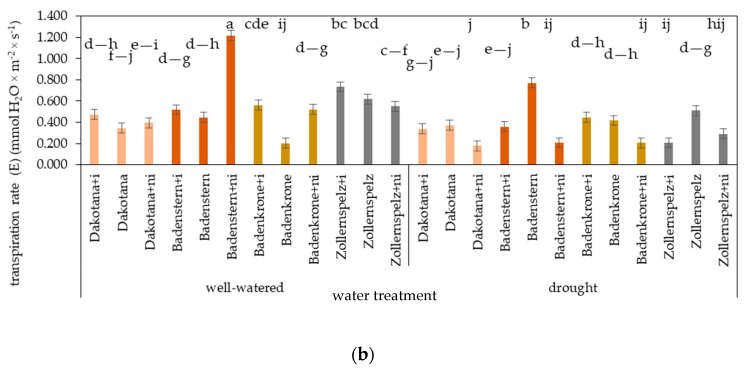
Effects of mycorrhizal inoculation (+i—with *G. irregulare* inoculation, +ni—natural inoculation) in wheat varieties under two water regimes (well-watered, drought) on: (**a**) photosynthesis rate (CO_2_ assimilation)—A (µmol CO_2_ m^−2^ s^−1^); (**b**) transpiration rate—E (mmol H_2_O m^−2^ s^−1^). Variety without +i/ni—grown in sterile soil. Letters a–j indicate statistically different mean values at *p* < 0.05.

There were significant interactions between water regimes, varieties and mycobiome composition, and the efficiency of photosynthesis measured during drought stress (Table 1, Figure 4). 

In well-watered conditions, both AM inoculation and natural inoculation significantly increased CO_2_ assimilation in the plants. Significantly increased CO_2_ assimilation was found in the spelt variety ‘Badenkrone’ with AM inoculation, and was 61.8% higher in comparison to ‘Badenkrone’ plants grown in the sterile soil. Under drought conditions, AM inoculation and natural inoculation resulted in decreased CO_2_ assimilation in the plants, with the exception of ‘Badenkrone’ with AM inoculation, which was at the same level as non-inoculated plants. Photosynthesis measurements under drought stress showed that the highest CO_2_ assimilation occurred in ‘Badenstern’ plants grown in sterile soil (Figure 4a).

The significant impact of water regime, variety and mycorrhizal inoculation on transpiration rate (E) was also recorded (Figure 4b). In well-watered conditions, the greatest transpiration rate was observed in ‘Badenstern’ plants grown with natural inoculation, and exceeded the rates observed for plants growing in the sterile soil and with AM inoculation by 63.4% and 57.4%, respectively. Under drought stress, AM inoculation and natural inoculation decreased the transpiration rate. In drought conditions, the greatest transpiration rate was observed in ‘Badenstern’ plants grown in sterile soil.

**Figure 5:**
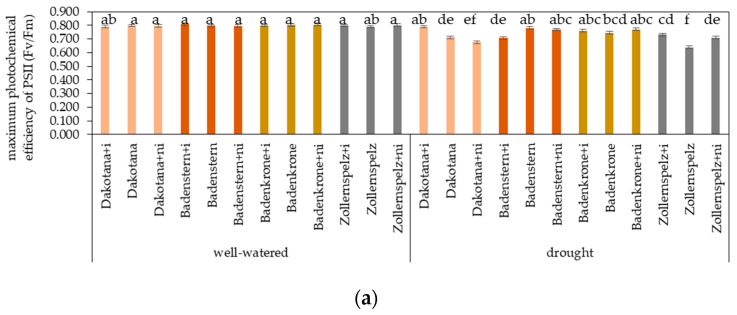

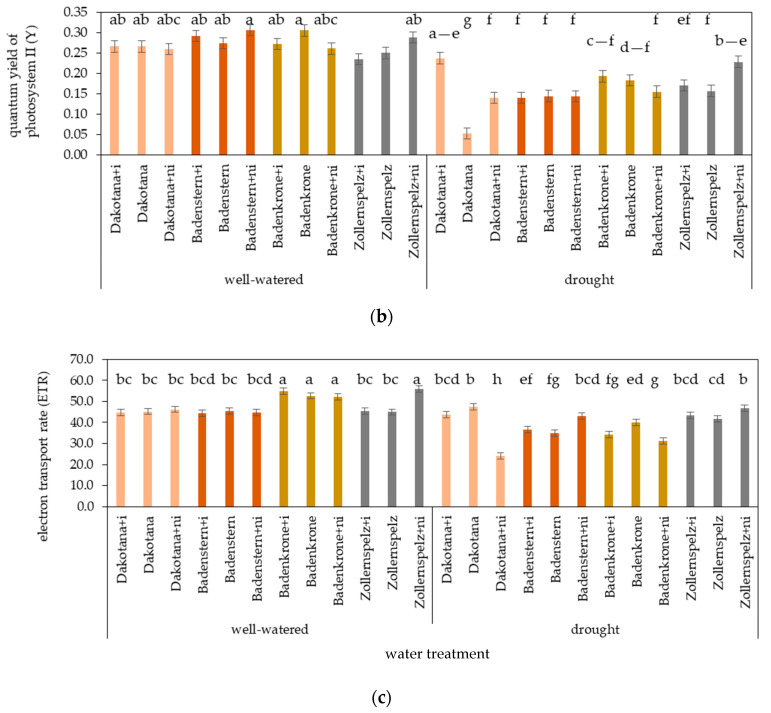
Effects of mycorrhizal inoculation (+i—with *G. irregulare* inoculation, +ni—natural inoculation) in wheat varieties under two water regimes (well-watered, drought) on the parameters of chlorophyll fluorescence (non-nominated units): (**a**) maximum photochemical efficiency of PSII (Fv/Fm), (**b**) quantum yield of photosystem II (Y) and (**c**) electron transport rate (ETR). Variety without +i/ni—grown in sterile soil. Letters a–h indicate statistically different mean values at *p* < 0.05.

Based on the analysis of chlorophyll fluorescence, there was a significant effect of water treatment, variety and mycorrhizal inoculation on the parameters measured after the plants had adapted to darkness: maximum photochemical efficiency of PSII (Fv/Fm) and both parameters measured under luminescence: quantum yield of PSII (Y) and electron transport rate (ETR) (Figure 5). 

The photosynthetic performance of the spelt and common wheat varieties was assessed by the maximum quantum efficiency of the PSII indicator. Under control conditions, this was observed to be 0.800, which suggests that the process was correctly performed. Under drought conditions, the greatest Fv/Fm values were observed in the common wheat variety ‘Dakotana’ and the spelt variety ‘Badenkrone’ with AM inoculation, ‘Badenstern’ plants grown with natural inoculation and in sterile soil and ‘Badenkrone’ with natural inoculation. Significantly lower Fv/Fm parameter values were recorded in ‘Zollernspelz’ plants grown under drought conditions and grown in sterile soil, and the common wheat variety ‘Dakotana’ with natural inoculation. AM inoculation significantly increased PSII efficiency in ‘Zollernspelz’ in comparison to plants grown in the sterile soil (Figure 5a).

During the drought measurements, significantly decreased quantum yield of photochemical reaction (yield parameter) values were observed in comparison to the well-watered treatment. AM inoculation of ‘Dakotana’ produced a significant increase in the Yield value in comparison to plants grown in sterile conditions and with natural inoculation, by 79.0 and 41.7%, respectively. Under well-watered and drought conditions, an insignificant increase in Yield parameters were found in ‘Zollernspelz’ with natural inoculation, although greater differences were observed under drought conditions, and the increase in the value in relation to plants grown in sterile conditions and with AM inoculation, under drought stress, was 30.4 and 26.1%, respectively (Figure 5b).

The highest ETR parameter value was found in spelt plants ‘Badenkrone’ and ‘Zollernspelz’ with natural inoculation, under well-watered conditions. Under drought stress, natural inoculation caused a significant increase in the ETR value in ‘Badenstern,’ in comparison to the plants grown in the sterile soil, while the effect was the opposite in ‘Dakotana’ and ‘Badenkrone’ (Figure 5c).

**Figure 6:**
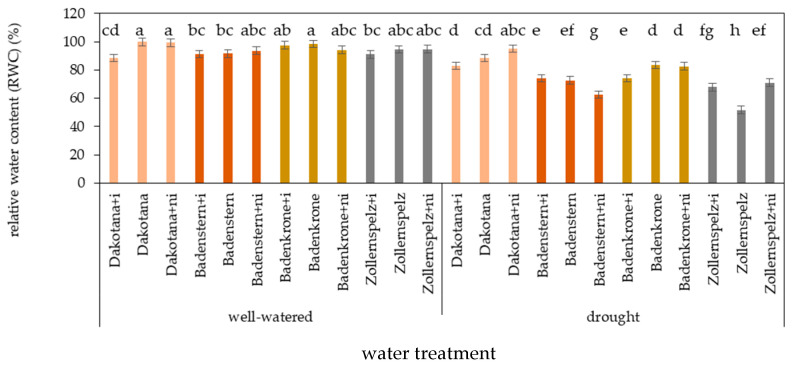
Relative water content (RWC: %) in leaves of wheat varieties inoculated with mycorrhiza (+i—with *G. irregulare* inoculation, +ni—natural inoculation) under well-watered and drought conditions. Variety without +i/ni—grown in sterile soil. Letters a–h indicate statistically different mean values at *p* < 0.05.

Under drought stress, the highest RWC value was observed in common wheat, and in the spelt wheat variety ‘Badenkrone’ (Figure 1). The RWC parameter measured in the leaves of the tested variety was influenced by water regime and microorganism inoculation factors (Table 1, Figure 6). 

Drought significantly decreased RWC in the leaves of all tested varieties with AM inoculation, except for ‘Dakotana’ with natural inoculation. Moreover, RWC values were comparable with the same treatment under well-watered conditions. Under drought conditions, natural inoculation significantly increased RWC by 26.8 p.p. in the spelt variety ‘Zollernspelz,’ in comparison to the treatment with no microorganisms (sterile growing conditions). The opposite effect was observed in the spelt variety ‘Badenstern,’ where a decrease of 13.7 p.p. was observed (Figure 6). 

**Figure 7:**
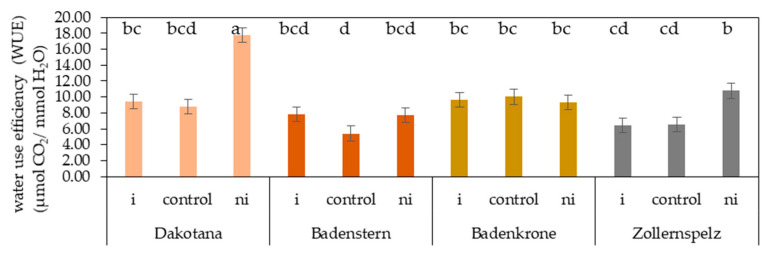
Effects of mycorrhizal inoculation (+i—with *G. irregulare* inoculation, +ni—natural inoculation) in wheat varieties on water use efficiency (WUE) (µmol CO_2_/mmol H_2_O). Control denotes variety without +i/ni—grown in sterile soil; letters a–d indicate statistically different mean values at *p* < 0.05.

Water use efficiency (WUE) of the spelt and common wheat varieties was influenced by the microorganism inoculation factor. The highest value was recorded in ‘Dakotana’ grown with natural inoculation; the increase in WUE in comparison to sterile growing conditions (without microbiota) and AM inoculation was 50.7 and 46.9%, respectively. Similarly, a significant increase in the WUE value from natural inoculation was observed in ‘Zollernspelz,’ and the difference with plants inoculated with AM and plants in the sterile soil was 40.5 and 39.6%, respectively (Figure 7).

### 2.3. Dry Matter Accumulation in the Parts of Plant

During water stress, irrespective of microorganism inoculation, the common wheat variety ‘Dakotana’ showed the lowest dry matter accumulation in terms of weight of aboveground biomass (stalk and spikes), while the greatest plant and stalk weights were found in ‘Badenstern’ and ‘Zollernspelz’ (Figure 1). Under drought conditions, plants grown either with AM inoculation or in a sterile soil had the greatest plant and stalk weights, while the lowest dry matter accumulation values were recorded in plants with natural inoculation, irrespective of the varieties studied.

**Figure 8:**
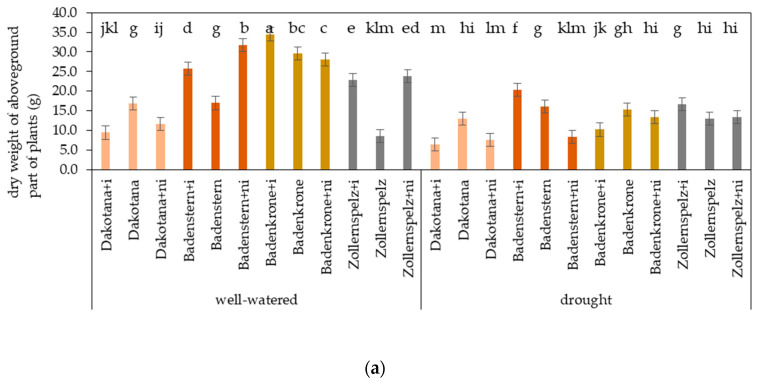

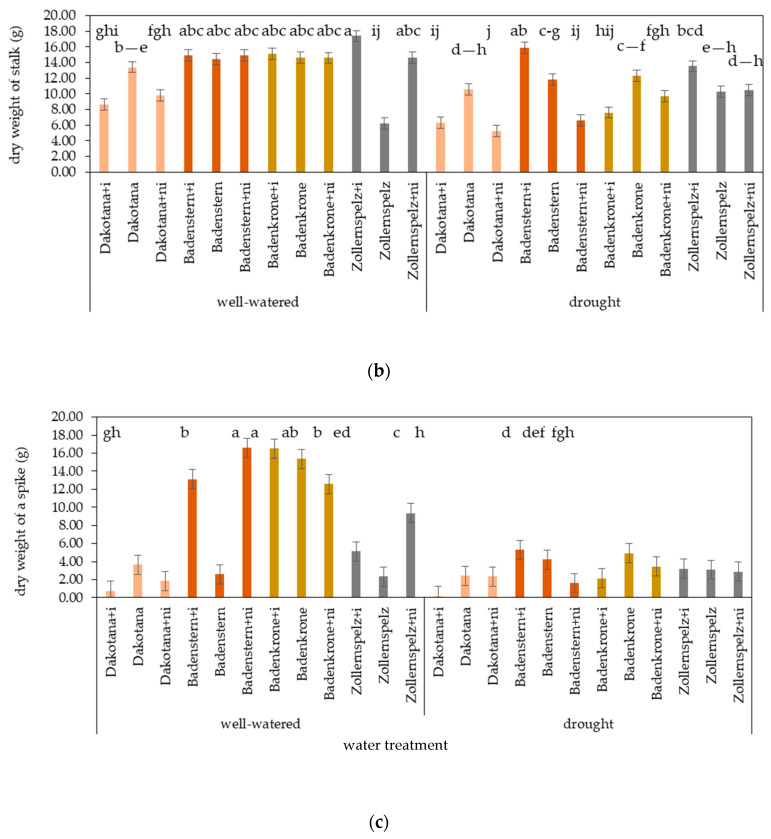
Effects of mycorrhizal inoculation (+i—with *G. irregulare* inoculation, +ni—natural inoculation) in wheat varieties under two water regimes (well-watered, drought) on dry weight of: (**a**) aboveground part of plants (Ab), (**b**) stalk (Sk) and (**c**) spike (Sp) (g m^−2^). Variety without +i/ni—grown in sterile soil. Letters a–m indicate statistically different mean values at *p* < 0.05.

In well-watered conditions, irrespective of inoculation status, the spelt wheat variety ‘Badenkrone’ was significantly heavier (by 18.1 g dry weight) than the common wheat variety ‘Dakotana,’ and was heavier by 5.96 and 12.36 g compared to ‘Badenstern’ and Zollernspelz,’ respectively. Under water stress, the same effect was observed with ‘Dakotana,’ which amounted an average of 9.01 g, while ‘Badenstern’ and ‘Zollernspelz’ were on the same level at 14.9 and 14.4 g, whereas ‘Badenkrone’ dry weight of aboveground part of plant amounted 12.9 g (Figure 1). In general, there were significant differences in plant dry weight between the tested varieties between non-inoculated (sterile), AM inoculated and natural inoculation in the two water regimes. Drought stress significantly decreased dry matter accumulation for all varieties, except for ‘Badenstern’ in the non-inoculated treatment where the dry weight amounted 16.1 and 16.9 g under drought and well-watered conditions, respectively. However, under drought conditions, AM inoculation led to the greatest increase in dry weight of aboveground biomass in ‘Badenstern,’ while under well-watered conditions the greatest values were recorded in ‘Badenkrone.’ Under drought stress, AM inoculation significantly decreased plant dry weight in ‘Badenkrone,’ as well as in common wheat, while it significantly increased dry weight in ‘Badenstern’ and ‘Zollernspelz’ by 20.7 and 22.2%, respectively (Figure 8a). A similar effect from microorganism inoculation and water regime was observed in regard to stalk dry weight. The greatest stalk dry weight values were found in ‘Badenstern’ and ‘Zollernspelz’ with *G. irregulare* inoculation (Figure 8b). The stalk dry weight of ‘Badenkrone’ was significant greater (by 4.14 g) than ‘Dakotana’ under well-watered conditions, irrespective of inoculation status, while under water stress ‘Badenstern’ and ‘Zollernspelz’ showed the greatest stalk dry weights—greater by 4.08 and 4.08 g, respectively in comparison to common wheat ‘Dakotana.’ Spike dry weight decreased in ‘Dakotana’ and ‘Badenkrone,’ both with AM inoculation and under water stress. Under well-watered conditions, the same was observed for common wheat, whereas ‘Badenkrone’ with AM inoculation and ‘Badenstern’ with natural inoculation exhibited the greatest spike dry weight values of 16.52 and 16.58 g, respectively (Figure 8c). Irrespective of microorganism inoculation status, ‘Badenkrone’ was observed to have the greatest spike dry weight under well-watered conditions, while under drought conditions, the spike dry weight of all the tested spelt varieties were at the same level, amounting to approximately 3.43 g; significantly higher than common wheat (1.65 g) (Figure 1).

### 2.4. Root Growth and Dry Matter Accumulation in the Roots

During water stress, irrespective of inoculation status, ‘Dakotana’ exhibited the lowest dry weight and root length values. A greater average root dry weight value was found in ‘Zollernspelz’ compared to the other spelt varieties, while the longest root was observed in ‘Badenstern’ (Figure 1). Regardless of variety, plants with natural inoculation grown under drought conditions showed the lowest root dry weight, length and volume. AM inoculation increased root length and volume, while the greatest root weight was observed in plants grown in sterile soil.

**Figure 9:**
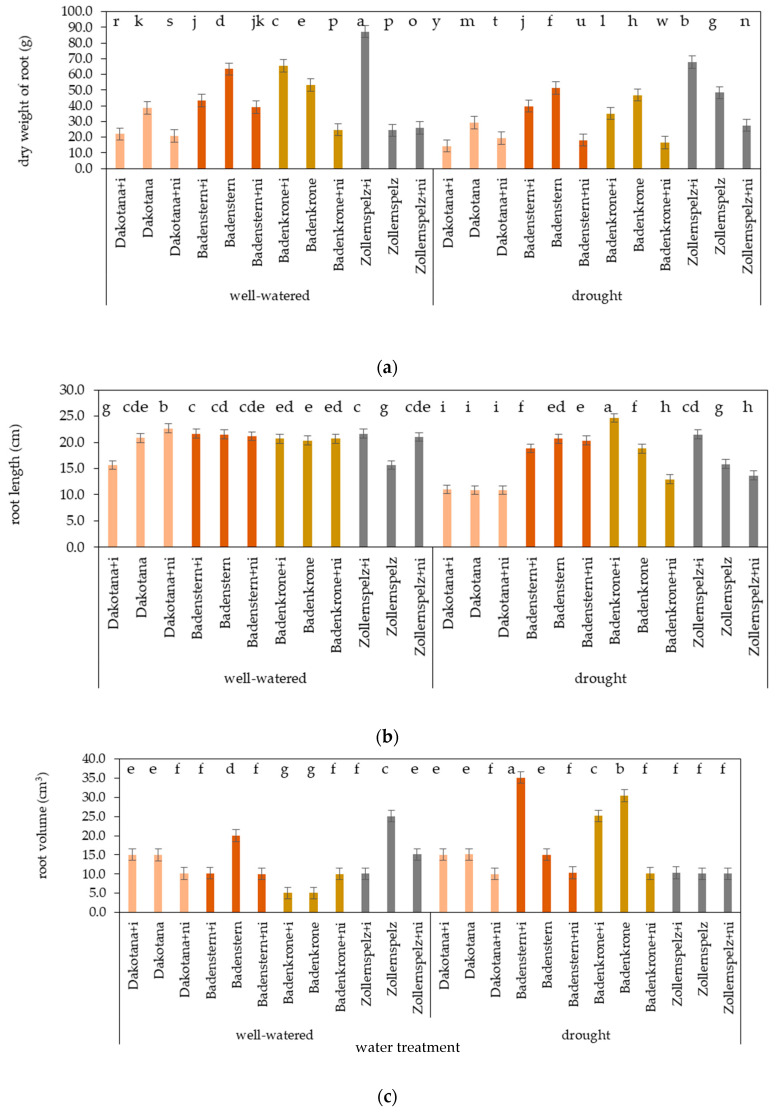
Assessment of wheat variety root growth according to mycorrhizal inoculation (+i—with *G. irregulare* inoculation, +ni—natural inoculation) under well-watered and drought conditions: (**a**) root dry weight (g), (**b**) root length (cm) and (**c**) root volume (cm^3^). Variety without +i/ni—grown in sterile soil; Letters a–y indicate statistically different mean values *p* < 0.05.

**Figure 10:**
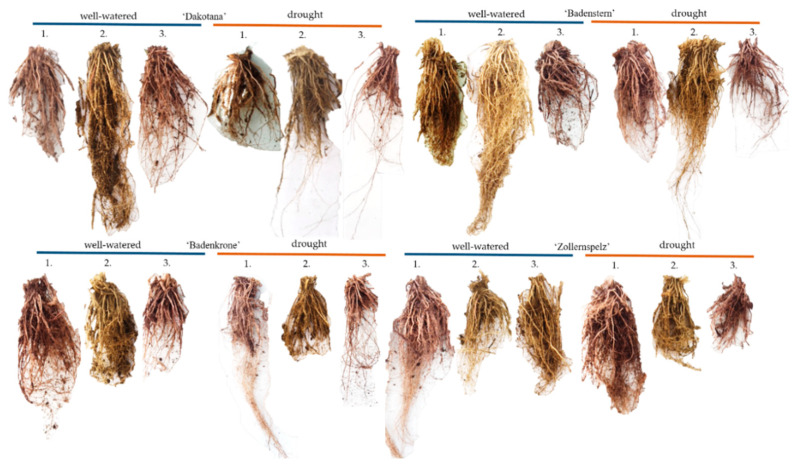
Effect of common wheat and spelt wheat variety root growth according to mycorrhizal inoculation (1. with *G. irregulare* inoculation, 2. grown in sterile soil and 3. with natural inoculation) under well-watered and drought conditions.

There were significant interactions between water regimes, varieties and mycobiome composition on root dry weight under well-watered and drought conditions (Table 1, Figure 9 and Figure 10). 

The greatest root dry weight value was found in ‘Zollernspelz’ plants with AM inoculation, both under well-watered and drought regimes. Similar to the dry matter accumulation, root weight decreased when AM inoculation was used, or when ‘Dakotana’ and ‘Badenstern’ plants were grown with natural inoculation under well-watered conditions. A similar decrease was also observed under drought conditions with ‘Badenkrone’ (Figure 9a). In general, there was significant difference in root dry weight between the spelt and common wheat varieties, regardless of inoculation status, while the greatest root dry weight was found in ‘Badenstern’ plants (48.6 g). The differences in common wheat, ‘Badekrone’ and ‘Zollernspelz’ were 21.4, 0.7 and 2.7 g, under well-watered conditions, respectively. Under drought conditions, the greatest root dry matter accumulation was observed in ‘Zollernspelz,’ which was 55.9, 23.8 and 31.5% greater than in ‘Dakotana,’ ‘Badenstern’ and ‘Badenkrone,’ respectively. 

Root length was also differentiated by variety, mycorrhizal inoculation and water regime. The longest roots were found under drought conditions in ‘Badenkrone’ with AM inoculation, and the increase in length in relation to the same variety and treatment under well-watered conditions was 4.4 cm. The smallest root length was observed in common wheat, with either AM inoculation in sterile conditions or with natural inoculation (Figure 9b). The best performance was found in ‘Badenstern,’ both under drought and well-watered conditions, irrespective of inoculation status. Under drought conditions, we found the greatest decrease in root length occurred in the common wheat variety ‘Dakotana,’ 9.0, 7.9 and 6.1 cm less than ‘Badenstern,’ ‘Badenkrone’ and ‘Zollernspelz,’ respectively (Figure 1).

The best root growth was obtained in ‘Badenstern’ with AM inoculation under drought conditions; root volume was also largest in this variety compared to all varieties grown under well-watered conditions. In addition, greater root volume values were observed in ‘Badenkrone,’ both inoculated and not inoculated (Figure 9c). Under well-watered conditions, and irrespective of inoculation status, the greatest root volume was found in ‘Zollernspelz,’ although under drought conditions it was the lowest. ‘Badenkrone’ and ‘Badenstern’ varieties exhibited the highest root volumes (Figure 1).

**Figure 11:**
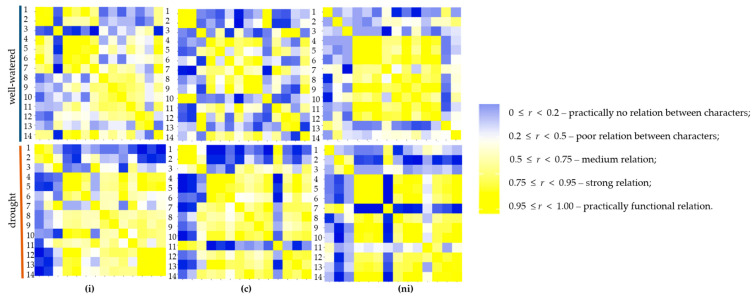
Heat map based on the pairwise Pearson correlation coefficients between agronomic traits (1. dry weight of roots (R), 2. length (l), 3. root volume (v), 4. dry weight of aboveground part of plants (Ab), 5. spike (Sp), 6. stalk (Sk), 7. root colonization (rc)) and physiological parameters (8. maximum photochemical efficiency of PSII (Fv/Fm), 9. quantum yield of photosystem II (Y), 10. electron transport rate (ETR), 11. transpiration rate (E), 12. photosynthetic rate (A), 13. water use efficiency (WUE), 14. relative water content (RWC)) in different mycorrhizal inoculation (i) with *G. irregulare* inoculation, (c) grown in sterile soil, (ni) with natural inoculation under well-watered and drought conditions. A darker blue color indicates a stronger negative correlation, a darker yellow color indicates a stronger positive correlation (legend presents detailed correlation coefficient).

**Figure 12:**
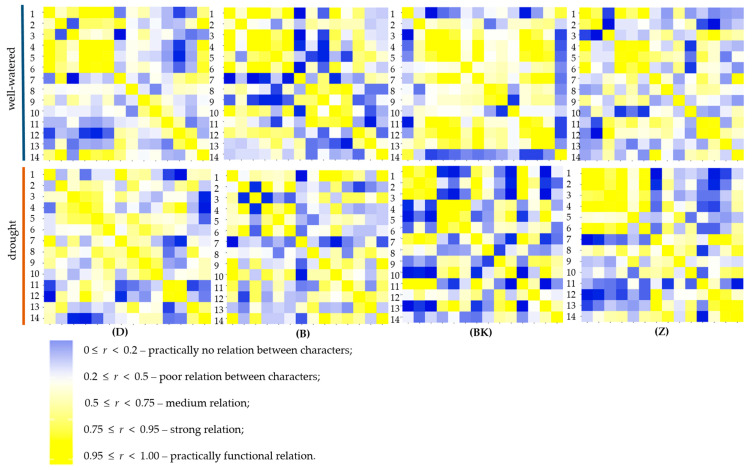
Heat map based on the pairwise Pearson correlation coefficients between agronomic traits (1. dry weight of roots (R), 2. length (l), 3. root volume (v), 4. dry weight of aboveground part of plants (Ab), 5. spike (Sp), 6. stalk (Sk), 7. root colonization (rc)) and physiological parameters (8. maximum photochemical efficiency of PSII (Fv/Fm), 9. quantum yield of photosystem II (Y), 10. electron transport rate (ETR), 11. transpiration rate (E), 12. photosynthetic rate (A), 13. water use efficiency (WUE), 14. relative water content (RWC)) in the common wheat variety ‘Dakotana’ (D), and spelt wheat varieties ‘Badenstern’ (B), ‘Badenkrone’ (BK) and ‘Zollernspelz’ (Z) under two water regimes (well-watered, drought). A darker blue color indicates a stronger negative correlation, a darker yellow color indicates a stronger positive correlation (legend presents detailed correlation coefficient).

Pearson’s correlation heatmap between agronomic traits and physiological parameters of common wheat and spelt within three different mycorrhizal inoculation treatment under well-watered and drought conditions are shown in Figure 11 (Supplementary Materials). It was remarkable that in plants with natural inoculation under drought most of the productive traits (dry weight of aboveground part of plants, spike and stalk) were positively correlated with maximum photochemical efficiency of PSII (Fv/Fm), quantum yield of photosystem II, electron transport, water use efficiency and relative water content. A strong significant correlation was found between dry weight of Ab and Sp (*r* = 0.98, *p* < 0.001), as well as between Sk (*r* = 0.94, *p* < 0.001). The quantum yield of photosystem II was significantly strong correlated with electron transport rate (*r* = 0.90, *p* < 0.001), but also with relative water content (*r* = 0.83, *p* < 0.01). The same relations were found in plants with mycorrhizal inoculation with *G. irregulare* and grown in sterile soil. On the other hand, dry weight of roots showed a negative correlation with the majority of agronomic and physiological parameters; especially significant negative correlation was found with (Fv/Fm) (*r* = −0.77, *p* < 0.01). The same relations were observed in other mycorrhizal treatment both under well-watered and drought conditions. It was remarkable that dry weight of roots was strongly related to root colonization (*r* = 0.91, *p* < 0.001) only in plants with mycorrhizal inoculation with *G. irregulare* under drought stress, while in well-watered conditions contrarily the relation was highly negative (*r* = −0.96, *p* < 0.001). In the case of the wheat and spelt varieties tested (Figure 12), such strong dependencies between trait as in the mycorrhizal treatment were not found. Interestingly ‘Badenstern’ spelt was the only variety with strong relation between dry weight of roots (*r* = 0.79, *p* > 0.001) and quantum yield of photosystem II (*r* = 0.77, *p* < 0.001) and electron transport rate (*r* = 0.80, *p* < 0.001) under drought stress. The quantum yield of photosystem II was found highly positively correlated with the relative water content (*r* = 0.92, *p* < 0.001). In common wheat ‘Dakotana,’ highly significant correlations were observed between the dry weight of roots and root colonization (*r* = 0.93, *p* < 0.001) in drought conditions, whereas under well-watered the root traits showed a reverse manner, and even highly negative relations between root volume and root colonization were observed (*r* = −0.99, *p* < 0.001) (Tables S1–S7).

### 2.5. Identification and Diversity of Fungi Isolated from Internal Root Tissues and Rhizosphere

**Figure 13:**
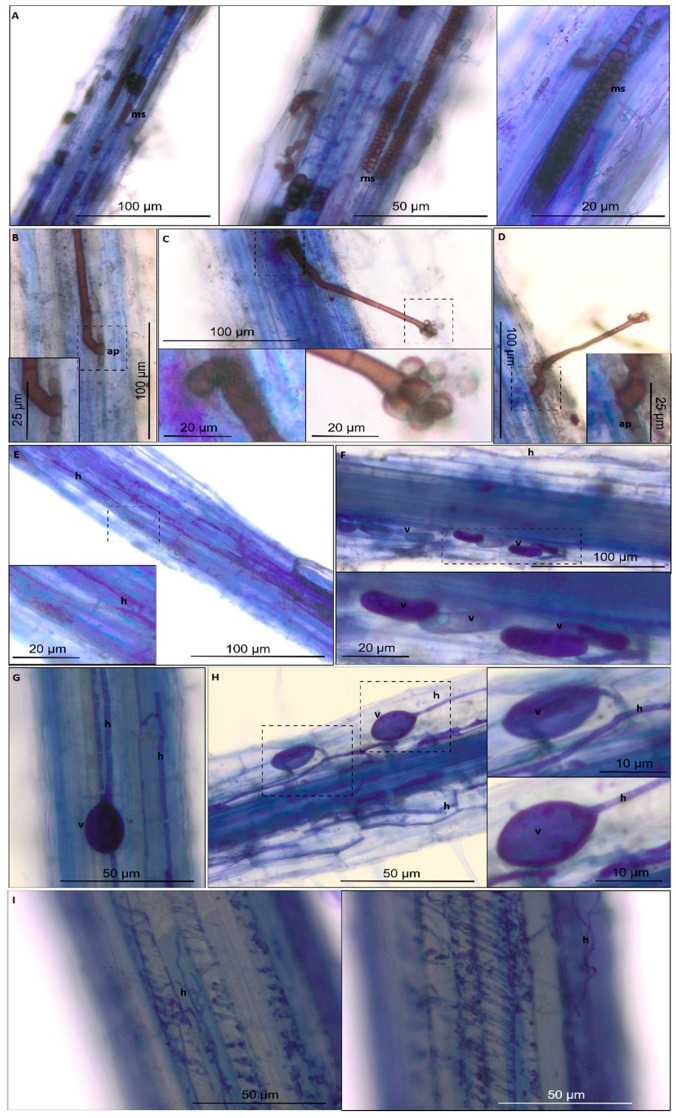
Selected micrographs taken with light microscopy. Panel (**A**) shows variety ‘Zollernspellz’ with natural inoculation grown under drought conditions, (**B**–**D**): ‘Badenkrone’ with natural inoculation grown under drought conditions, (**E**,**F**): ‘Badenstern’ without inoculation grown under well-watered conditions, (**G**,**H**): ‘Badenstern’ with natural inoculation grown under well-watered conditions, (**I**): ‘Badenkrone’ with mycorrhizal inoculation grown under drought conditions. Abbreviations: (v) vesicle, (h) hyphae, (ms) microsclerotia and (ap) apresorium. Dotted lines marked magnifications locations.

Analysis showed that the roots in all tested variants of wheat were colonized by fungi. Nevertheless, the colonization percentage varied between groups and ranged between 59% to 100% (Figure 3). Microscopy observations showed that fungal hyphae grew into the roots (Figure 13A–I), and fungi were formed as oval (Figure 13G,H) and longitudinal (Figure 13F) shaped vesicle structures. 

Moreover, fungal macroslerotia was observed in the root cells of ‘Zollernspellz’ with natural inoculation under drought conditions (Figure 13A). Fungi from *Periconia* spp. [22,23] can be seen in the root cells of ‘Badenkrone’ with natural inoculation under drought conditions in Figure 13B–D.

A total of 128 fungal isolates were cultured from the rhizosphere and the internal parts of the roots of the studied varieties across all greenhouse growth conditions. Fungi were more abundant in the common wheat ‘Dakotana’ variety (40 isolates) than in the spelt wheat varieties ‘Badenstern,’ ‘Badenkrone’ and ‘Zollernspellz,’ where the number of isolates was 38, 24 and 26, respectively. If the isolates were grouped according to plant growth conditions, the greatest number (22) was found in plants that were grown with natural inoculation, followed by mycorrhizal inoculation and without mycorrhizal inoculation under well-watered conditions. Fungal occurrence was least (15 isolates) in plants that were grown under drought conditions with mycorrhizal inoculation. Molecular analysis resulted in the identification of 37 species, representing the following genera: *Albifimbria*, *Alternaria*, *Apodus*, *Arthopyrenia*, *Zopfiella*, *Curvularia*, *Diaporthe*, *Fusarium*, *Gaeumannomyces*, *Magnaporthiopsis*, *Marasmius*, *Microdochium*, *Mucor*, *Periconia*, *Rhizoctonia*, *Setophoma* and *Trichoderma*. Shannon’s biodiversity (H) and evenness (E) indices showed that the greatest species diversity (H′ = 2.58) and relatively well distribution (E = 0.93) was in ‘Zollernspellz,’ followed by ‘Dakotana’ (H′ = 2.52, E = 0.87) (Table 2). The Shannon diversity index showed that the plants that grew with natural inoculation under well-watered conditions had the greatest species diversity (H′ = 2.54), whereas the plants that grew with mycorrhizal inoculation under drought conditions had the lowest species diversity values (H′ = 1.41). These conditions were also characterized by the least homogeneous distribution of species (E = 0.79) (Table 3). Species diversity was quite similar in all wheat varieties—greater under well-watered conditions; Shannon index values ranged from 1.55 in ‘Badenstern’ with mycorrhizal inoculation, to 2.04 in ‘Dakotana’ with natural inoculation, growing under well-watered conditions. Values were less in drought conditions and ranged from 0.56 in ‘Dakotana’ without inoculation to 0.64 in ‘Badenstern’ with mycorrhizal and natural inoculation and ‘Badenkrone’ with mycorrhizal inoculation (Table 4).

## 3. Discussion

A considerable body of research suggests that a diverse array of microbiota colonize plant organs and tissues, including the roots in the rhizosphere (e.g., Banach et al., Toubal et al.) [24,25]. Moreover, evidence suggests that a greater abundance of endophytes are found in the plant roots and other underground tissues than in the aboveground plant organs [26]. Endophytic colonization may be controlled by many factors, such as host species, plant organs, geographic locality and seasonality) [27]. Numerous studies have investigated wheat growth promotion by microbes, which include root-inhabiting fungal species, in different abiotic stress conditions, such as cold stress [28], drought and heat stress [29], acidic and alkalinity stress [30] or salinity stress [31]. Although our knowledge of this interesting group of endophytes is constantly expanding, only one recent study has explored how the structures of fungal communities in wheat and spelt wheat are affected by drought stress, but no information is available as to how they affect plant growth. To date, only one report [32] appeared in plant breeding, no practical application of genotypic differences in plant response to mycorrhizae has been reported [33]. In this study, it was assumed that the composition of fungal communities in the root endosphere in the spelt wheat varieties differed from the common wheat varieties, as would their response to drought stress. We assumed that one of the studied varieties would exhibit greater drought resistance, while mycorrhizal inoculation would enhance plant growth through alleviating soil stress.

Drought causes cell wall leakage and water uptake, which adversely affect crop growth. In assessing the physiological state of plants under drought stress, gas exchange assessments can provide valuable information as to the uptake and transport of water and nutrients, which has been reported to correlate with gas exchange [34]. Li et al. [35] demonstrated that AM fungi improved gas exchange in barley plants under drought stress, evidenced by greater photosynthetic and transpiration rates than in corresponding non-mycorrhizal barley plants. Our results showed that AM and natural inoculation resulted in increased A and E values in well-watered conditions, but in decreased values under drought stress. However, some studies have shown that during soil drying, AM root colonization could increase stomatal conductance and maintain higher gas exchange rates than in non-mycorrhizal plants [36,37], which is explained by the fact that AM plants typically exhibit greater photosynthetic rates than non-AM plants due to the demand for carbon compounds from the fungi [38]. In our study, only ‘Badenkrone’ with AM inoculation did not show significantly decreased A and E values. Although spelt wheat is commonly reported to be more tolerant (hardy) to environmental stresses, the physiological state of plants under drought stress varied between the varieties studied here; ‘Badenstern’ showed improved gas exchange under drought stress, irrespective of inoculation status. Silva [39] reported that spelt is naturally more enriched in macronutrients and micronutrients than wheat and also has a better growth efficiency under drought conditions, thus, this may explain our results. In addition, a photoprotective response was observed in ‘Badenstern’ plants grown in sterile soil due to greater photosynthetic and transpiration rates than in the other wheat varieties.

Changes in the physiological state of plant tissues can occur due to water losses. New methods for the detection of such changes through the use of chlorophyll fluorescence has shown a significant decrease in photosynthetic activity with the reduction of photochemical reactions that occur in PSII under drought conditions. Chlorophyll fluorescence indices, such as Fv/Fm, Y and ETR, depend on water content level, variety and inoculation treatment used. A similar effect under drought conditions was obtained in studies with *T. aestivum* L. ‘Ilona’ [40], with the exception of the Fv/Fm parameter, which was almost unaffected. Our results showed no differences between common wheat varieties with AM inoculation under well-watered conditions, while both sterile (non-inoculated plants) and those growing with natural inoculation exhibited significantly decreased Fv/Fm parameter values. Under drought conditions, a similar effect was observed in ‘Badenkrone’ and ‘Zollernspelz,’ although AM inoculation slightly increased the Fv/Fm value under drought conditions. Inoculation of ‘Dakotana’ with AMF in our study increased the quantum Yield of photosystem II (Y parameter) under water deficits, which is in agreement with studies on spring wheat *Triticum aestivum* ‘1110′ by Zhou et al. [37]. This is also consistent with the finding that AMF maintain the integrity and stability of PSI and PSII, and thus, protects the photosynthetic apparatus of wheat plants under drought stress [41]. Irrespective of microorganism inoculation, our study showed that Fv/Fm and E values were greater in spelt wheat varieties ‘Badenstern’ and ‘Badenkrone,’ than in common wheat, which is consistent with Konvalina et al. [42], who showed that spring spelt wheat *T. spelta* ‘Kew’ was more predisposed to drought tolerance than comparable wheat varieties. Those authors suggested that modern bread wheat varieties may be more affected by drought than wheat landraces.

The magnitude of the effect of water stress on photosynthetic traits differed between varieties. Although ‘Dakotana’ showed decreased photosynthesis rates, the reduction in water content in the leaves of plants grown with natural inoculation was insignificant and was comparable to well-watered conditions. This response occurred earlier in the common wheat variety ‘Dakotana’ than in the spelt varieties, which would suggest that common wheat is more resistant to dehydration than spelt wheat. Bandurska et al. [43] also observed a slight reduction in water content in barley leaves under water deficits, thereby indicating the rapid closure of stomata. Similar insights were reported by Pelleshi et al. [44] in research on two maize varieties; the relationship between net CO_2_ uptake and RWC showed that a decrease in photosynthetic rate occurred before any changes in RWC, and RWC varied only when net CO_2_ uptake was lower than 70% of the control. Similarly, Wu et al. [45] observed lower transpiration rates with a molybdenum treatment, which helped maintain an elevated water status in wheat under drought stress. In contrast to our results, some recent studies (e.g., Zivcak et al.) [40] have observed direct correlations between leaf water status and CO_2_ assimilation, and the PSII electron transport rate. Similar to our results, Boutraa et al. [46] using RWC to screen for drought tolerance, considered one of the wheat cultivar Sindy-1 and Sindy-2 as drought-tolerant, although the effects of water stress on other growth functions indicated that Sindy-1 was not. In our research, we noted a similar finding with ‘Dakotana’; a positive reaction to AMF inoculation was observed by greater efficiency and Yield of PSII in the photosynthetic apparatus in the leaves. Improving this function did not contribute to enhanced drought resistance since RWC and WUE values were lower than those in plants grown in sterile conditions.

The results obtained in this study showed a decrease in WUE values in wheat varieties as a result of water stress, similar to previous research on wheat [47,48], millet, barley or sorghum [49]. The common wheat variety ‘Dakotana’ grown with natural inoculation took up the least amount of water of all varieties. Moreover, microorganisms in the soil also increased WUE values in spelt wheat ‘Zollernspelz.’ In rice intensification studies, Zhao et al. [50] showed that one of the systems studied reduced water consumption and increased WUE, which the authors explained was beneficial for improving soil fertility because of the effects on soil microbial biomass. Moreover, Dodd and Ruiz-Lozano [51] indicated that soil biota may sustain crop yields despite decreased nutrient and water inputs, thereby improving crop resource use efficiency. The results from Li et al. [35] showed that *R. intraradices* (AMF) inoculation increased WUE values in barley plants and had more profound impacts on WUE in a root-hairless barley mutant grown under drought conditions. Our research did not show any changes in WUE in the wheat variety with mycorrhizal inoculation under drought conditions.

Assessment of drought tolerance in plants is difficult, since most resistant varieties are those that exhibit tolerance at multiple levels at the same time. In most cases, such evaluations are complex, using physiological and morphological methods [52]. Among the eight major crops (wheat, barley, corn, sorghum, soybean, oat, potato and sugar beet), wheat is the most sensitive to abiotic stresses involving drought [53]. Many researchers have confirmed that water stress can lead to growth reduction, which is reflected in dry weight values and other growth functions in cereal crops [43,54]. Boutraa et al. [46] indicated that while water stress affects most of the functions of plant growth, the effect depends on the level of water stress, the length of time to which the plant is subjected to water stress and the variety or plant species. Our research also indicates that it depends on the variety, and in this regard, the best performance (dry weight of aboveground parts of plant, spike and stalk) occurred in ‘Badenstern’ and ‘Zollernspelz,’ irrespective of inoculation status. In addition, inoculation with AMF fungi was observed to contribute to the increase in the above parameters in ‘Badenstern’ and ‘Zollernspelz,’ but led to a decrease in the same parameters in ‘Badenkrone’ and ‘Dakotana.’ Such reactions within varieties are consistent with other studies (e.g., Zhou et al. and Mobasser et al.) [37,55]. The reaction of varieties to AMF inoculation can be varied [55,56] and the positive effect of AMF can be attributed to increased water and nutrient-uptake by the external hyphae that may increase the contact between the soil and roots [57], penetrate soil pores inaccessible to root hairs in drying soils [58] when cavitation in drying soils blocks water movement from the soil to the roots [35]. 

Zhou et al. [37] also showed decreased aboveground biomass and grain yield in one wheat variety ‘Vinjett’ with AMF inoculation under drought conditions and suggested that this was caused by competition for assimilates between AMF and the host plant, as root-associated fungi use these assimilates as energy resources. Similarly, Borkowska [59] indicated that mycorrhizal association supplies the plants with water to protect the system against drought but at the same time they are very active “consumers” of the assimilates. AMF have been reported to increase shoot dry weight and grain yield in rice cultivars [60] or maize [61]. Until our study, the response of spelt wheat to AMF under drought conditions had not been addressed.

Researchers consider that an increase in root growth is an indicator of the ability of a plant to withstand water stress, and can also provide information to screen plant cultivars for drought tolerance [62]. It has been shown that wheat plants in arid areas can increase their root surface and improve plant water uptake [63]. In our research, both dry weight and root length decreased, while the root volume of wheat increased under drought conditions. This may indicate that roots stimulate hair density and proliferation to cope with drought stress. For example, root hairs have been shown to enhance the tolerance of barley plants to phosphorus (P) deficiency under water stress conditions [64]. In our study, ‘Badenstern’ was considered more tolerant to drought as it exhibited greater root length and volume than the other varieties studied under drought conditions. However, ‘Zollernspelz’ exhibited the greatest root dry weight values in comparison to the other studied varieties. Similarly, Xie et al. [65] noted that spelt has a large root system, and indicated that it could be a useful genetic resource for root system architecture enhancement in bread wheat, while seminal root number and total root length, in particular, are favorable for the improvement of yield potential, and should be incorporated into wheat ideotypes. Silva [39] showed that spelt is naturally more enriched in macronutrients and micronutrients than wheat and also has a better growth efficiency under drought conditions. In our study, the common wheat variety ‘Dakotana’ exhibited the lowest root growth parameters, which is consistent with the finding that modern bread wheat cultivars have a smaller root mass compared with landraces [66]. In addition, inoculation of the spelt variety ‘Badenkrone’ with AMF led to increased root length, yet produced an increased root volume in ‘Badenstern.’ In turn, inoculation of ‘Zollernspelz’ with AMF increased dry weight and root length. Older studies have shown that both above- and below-ground biomass growth increases in wheat plants inoculated with *G. fasciculatum* and *G. deserticola* [67], and *G. mosseae* [68] under drought conditions. In our study, ‘Badenstern’ inoculated with AMF under drought conditions increased aboveground dry matter content, while the root dry weight was less in the plants grown under sterile conditions. According to Ruiz-Lozano et al. [69], the low root dry weight can be associated with AMF symbiosis, which significantly improves the resistance of inoculated plants to drought by reducing the need for an increase in roots. Libault et al. [70] and Oldroyd and Downie [71] have pointed out that the morphology of root hairs and the release of compounds into the rhizosphere mediate the interaction of plants with beneficial soil microorganisms, such as AMF, which may help plants take up more water and nutrients under drought stress. In addition, a vigorous root system should produce abundant secretions that may help the reproduction of microbes [62]. Li et al. [35] suggested that both AMF (*R. intraradices*) and root hairs improve barley plant tolerance to drought stress. 

In recent years, the main use of mycorrhizal technology has been to improve growth in a number of micro-propagated horticultural crops. For example, mycorrhized strawberry plants grown under drought conditions have been found to have increased biomass accumulation rates (crowns and roots), and larger leaf area; the root system remained significantly larger in the AMF plants as the plants developed [59]. The use of mycorrhiza in agricultural crops has not been extensively investigated, although the work by Lehnert et al. [32] would suggest that mycorrhizal fungi could be used effectively in agriculture in the future and could be combined with new approaches, e.g., the use of genotypic variation with existing drought tolerance breeding programs to develop new drought tolerant varieties. Moreover, Dodd and Lozano [51] perceived the opportunity to develop microbial inoculants with a relatively modest financial outlay with the use of isolation, characterization and ultimate application that will lead this area of crop biotechnology in the future, especially at risk from problems of food security.

In our study, root colonization under drought stress conditions was significantly less than root colonization under well-watered conditions, which is in agreement with Lehnert et al. [32]. In our research, mean root colonization was 76.7% and 77.6% greater under drought conditions and well-watered conditions, respectively, compared to 35% (under drought) and 47% (well-watered) observed in Lehnert et al. [32]. In the latter study, the authors showed some evidence that the effective use of symbiosis depends on a balanced interaction between plant and fungi, as well as environmental conditions, rather than the level of root colonization by mycorrhizal fungi. In our research, typical mycorrhizal structures were detected in the roots both in the inoculated plants, and those grown on both sterile and non-autoclaved field soils. Some previous studies have described endophytes as wheat root-associated fungi of the common wheat—*T. aestivum* ssp. *vulgare* L. [72,73]—but only one study has focused on spelt wheat—*T. aestivum* ssp. *spelta* L. [9]. In Kuźniar et al. [9], fungal species diversity was greater in the common wheat variety ‘Hondia’ than in the spelt wheat variety ‘Rokosz,’ while in our research the ‘Zollernspellz’ variety was more species-rich than the common wheat variety. The species diversity of the microbiota that inhabit the root zone may be considerable given that none of the fungal species identified in Kuźniar et al. [9] were found in our study. Here, we have demonstrated that the wheat variety ‘Dakotana’ was the most species rich (40 isolates), while the spelt varieties contained, on average, 29 species (‘Badenstern’ contained 38 species). Drought decreased the number of isolates, especially in plants growing under drought conditions with mycorrhizal inoculation. Stress can indirectly affect symbiosis, although it seems to improve growth and physiological activity in both common wheat and spelt varieties. Interestingly, *G. irregulare* was able to survive in the roots of ‘Badenstern’ in our study, even under drought conditions.

## 4. Materials and Methods

### 4.1. Experimental Procedure

The experiment was conducted in a greenhouse at the Agronomy Department, Poznań University of Life Sciences. Experimental soil (0–20 cm depth) was collected from the field (52°48′ N, 16°82′ E) where both spelt and wheat were grown (Research and Education Center of Gorzyń, Złotniki Research Station, Poznań University of Life Sciences, Poland). The soil is classified as luvisol of light clay sand grade, shallowly deposited on a light clay that belongs to a good rye complex [74]; the soil is pH 5.4, with an average content of 120.6 mg P_2_O_5_ kg soil^−1^ (very high), 122.5 mg K_2_O kg soil^−1^ (high), with 0.59% organic matter. This experimental soil was used as the growth medium for the plants and was sieved through a 4 mm mesh, and was then sterilized by autoclaving at 120 °C for 1 h, and left for 2 days before the experiment started. Wheat seedlings (approximately 8 cm length) were collected from the experimental field at the Złotniki Research Station, were washed in running water and disinfected in potassium manganate (0.05% KMnO₄) for 1 min. Plastic polyethylene pots (7L) were filled with the growth medium, and two seedlings of equal length with the best growth performance were transplanted in each pot. Arbuscular mycorrhizal fungi (AMF) *R. irregularis* inoculum (2 mL/plant containing 2000 spores) were applied to the upper third of each pot designated for AMF treatment. Control pots instead received 2 mL of deionized water.

For the establishment of two water regimes, 50% of the containers (sterile and inoculated, and non-sterile and non-inoculated) were subjected to drought stress where the soil water content was maintained at 8% (approximately 36% field water capacity), while the remaining 50% of containers were maintained under well-watered conditions (15% water content equating to 70% field water capacity) throughout the entire experiment. To maintain the desired moisture content, the pots were regularly watered (250 mL H_2_O/pot every 48 h) and monitored daily with a probe (ThetaProbe, Eijkelkamp Penetrologger SN, Giesbeek, The Netherlands). Drought stress was initiated in the flowering phase (BBCH 65–69), by stopping watering of the plants. After 8 days of drought, the soil moisture content reached 5–8% *v/v*, and was available to the plants. At this stage, all the leaves had lost their vigor, and some of the leaf blades were curled.

In total, there were 24 (2 × 4 × 3) combinations, with four replicates for each combination (water regimes: well-watered (pots irrigated with deionized water) and drought (pots not irrigated in the flowering phase); three spelt varieties: *T. aestivum* ssp. *spelta* L.: ‘Badenstern,’ ‘Badenkrone’ (both ZG Raiffeisen eG, Karlsruhe, Germany) and ‘Zollernspelz’ (Saaten Union, Isernhagen, Germany); one wheat variety: *T. aestivum* ssp. *vulgare* L: ‘Dakotana’ (KWS Saat, Einbeck, Germany); three mycorrhizal levels: autoclaved soil inoculated with *R. irregularis* (*Glomus irregulare*), DAOM 197198 strain (Connectis, Agronutrition, Carbonne, France) (i), control (autoclaved soil), natural inoculation (non-autoclaved soil—microorganisms from the field) (ni)). The resulting 96 containers were set up in a completely randomized block design. The experiment was conducted in a greenhouse for a four-month period (from April to July) (16:8 h and 18–30 °C, 60–70% relative humidity) at the Agronomy Department, Poznań University of Life Sciences. All treatments were regularly fertilized using Florovit (5 mL/2 L H_2_O) and ammonium nitrate (1 g/pot) one month after the start of the experiment.

### 4.2. Parameters Measured during the Stress Period Induced in the BBCH 65–69 Phase

#### 4.2.1. Relative Water Content

Water content in leaves was estimated by measuring relative water content (RWC) according to Weatherly [75] as described by Bandurska [76].

Cut leaves were weighed (fresh matter, FM), the leaf samples were then placed in distilled water for 4 h under normal room light and temperature and the FM content (maintained at full turgor, TW) was measured. The samples were then dried in an oven at 70 °C for 24 h to determine dry weight (dry matter, DW). RWC (1) was calculated as follows:

(1) $$RWC=\frac{FM-DM}{TW-DM}\times 100$$

#### 4.2.2. Leaf Chlorophyll Fluorescence

Chlorophyll fluorescence was measured on a fully mature, healthy leaf using a portable modulated chlorophyll fluorometer (OS5p, Opti-Sciences, Inc., Hudson, NY, USA). The samples were light-adapted using photosynthetic active radiation (PAR) clips for 30 min before the parameters were measured, after the selection of the kinetic test mode. Protocol settings were adjusted based on the previous study by Sulewska et al. [77]. The following parameters were measured: *F*0—minimum fluorescence and *Fm*—maximum fluorescence to calculate the *Fv*/*Fm* parameter considered to be a ready indicator of plant photosynthetic performance (value of approximately 0.83–0.85 describes a healthy sample) [78]. The following formula for the maximum photochemical efficiency of PSII (*Fv*/*Fm*) (2) was used:

(2) $$Fv/Fm=\frac{Fm-F0}{Fm}$$

The parameter quantum Yield of Photosystem II (Y) was measured and used to calculate the electron transport rate (ETR) (3), according to the formula applied by Krall and Edwards [79]:

(3) ETR=Y×0.84×0.50×PPFD

where PPFD (60 µmol photons m^−2^ s^−1^) is the photosynthetic photon flux density incident on the leaf, 0.5 is a multiplication factor (as transport of a single electron requires the absorption of two light quanta) and 0.84 is the specific fraction of incident quanta absorbed by the leaf (ETR-factor) [80,81].

#### 4.2.3. Leaf Gas Exchange and Water Use Efficiency (WUE)

Net CO_2_ assimilation rate (photosynthetic rate: A) and leaf transpiration rate (E) were measured on the same fully mature leaf with a portable photosynthesis system (LCpro-SD, ADC BioScientific Ltd., Hoddesdon, UK) with a narrow leaf chamber (area: 5.8 cm^2^). During the period of measurements, CO_2_ concentration (reference CO_2_) in the leaf chamber was kept at 360 ppm, leaf chamber temperature was maintained at 25 ± 1 °C, the flow rate of air was kept at 200 µmol s^−1^ and ambient H_2_O concentration (Reference H_2_O) and PAR were adjusted automatically by a red-blue light-emitting diode (LED) light source (LCP Narrow Lamp, ADC BioScientific Ltd., UK). Water use efficiency (WUE) (4) was calculated according to the following equation:

(4) $$WUE=\frac{A}{E}$$

### 4.3. Parameters Measured during the BBCH 73–77 Phase

#### 4.3.1. Biomass Production

Dry matter accumulation was measured after the experimental period. All plants from each treatment were sampled. Plant stalks, spikes and roots were separately harvested. The adhering soil particles were washed off the roots with deionized water. Roots were immersed into a partially filled graduated cylinder and the resultant water displacement was recorded to determine the root volume. Stalk, spike and root dry weights were recorded after drying the samples at 70 °C for 48 h.

#### 4.3.2. Microscopic Observations of the Root Surface

The fungi method proposed by Phillips and Hayman [82] was used with slight modifications for the detection of colonized roots. Plant roots were collected and washed with deionized water. Ten fragments (1 cm long) were taken from the thinnest roots of each plant. Fragments were incubated in 5% KOH (Avantor Performance Materials Poland S.A.) for 24 h. Subsequently, KOH was removed and the material were washed three times with deionized water and incubated in 50% lactic acid (Avantor Performance Materials Poland S.A., Gliwice, Poland) for 1 h. The detection process was carried out with 0.1% trypan blue (Merck KGaA, Darmstadt, Germany, formerly Sigma, St. Louis, MO, USA) in lactoglicerol solution (1:1:1) of glycerol (Merck KGaA, Darmstadt, Germany, formerly Sigma, St. Louis, MO, USA): lactic acid: deionized water] for 2 h. All incubations were performed at room temperature. The observations were carried out using a light microscope (Olympus CX-41-1 with UC-30 camera, Olympus, Tokyo, Japan). For the estimation of colonized wheat roots, each 1 cm long fragment was observed under microscope to study mycelia, vesicles and arbuscules. Calculation of the percentage of root colonization (5) was carried out with the following formula:

(5) $$\%\;of\;root\;colonization=\frac{number\;of\;colonized\;roots\;fragment}{total\;number\;of\;examinated\;fragments}\times 100$$

#### 4.3.3. Fungi Isolation

Fungal isolates were obtained from the rhizosphere and from the internal root tissues of the wheat and spelt plants. Roots from two random plants from each treatment were removed and sectioned into 4–5 cm pieces before sterilization. The outer root layer, together with the rhizosphere, were mechanically separated by brushing. The surface of the tissues was sterilized with 70% ethanol, 0.5% active chlorine, and rinsed several times in sterile distilled water until no chlorine was discernible. Roots samples were cut with a sterile scalpel to produce 1 cm long sections. Non-sterile fragments, together with the rhizosphere and sterilized sections of the roots, were placed on Potato Dextrose Agar (PDA, Oxoid™ Thermo Fisher Scientific, Walthman, MA, USA) supplemented with an antibiotic. The PDA-Petri dishes were incubated at 22 °C until the appearance of mycelia. Emerging fungal colonies were repeatedly subcultured on PDA to obtain homogeneous cultures. Purified cultures were subsequently transferred to tubes containing Synthetischer Nährstoffarmer Agar (SNA; Nirenberg 1976) and stored at 4 °C for further study. At the same time, approximately 10–50 mg of mycelium was collected into Eppendorf tubes and stored at −20 °C until DNA isolation.

#### 4.3.4. Fungi Identification

Fungal isolates were identified by sequencing ITS regions 1 and 2 (ITS1 and ITS2) of the rRNA gene cluster and a fragment of the translation elongation factor 1-alpha (*Tef1*) gene, and also on the sequencing of the partial beta-tubulin 2 (*βtub*) gene, depending on the fungal genus. DNA was extracted from the homogenized mycelium in the liquid nitrogen of the isolates, which were grown previously on the PDA medium using Wizard^®^ Genomic DNA Purification Kit (Promega, Madison, WI, USA). The ITS1 and ITS2 regions of the rDNA gene cluster were amplified using primers ITS4 and ITS1F [31], the small unit region was amplified using primers NS1 and NS4 [83], the large unit region was amplified using primers LROR [84] and LR6 [85], while a fragment of the *tef1* gene was amplified using primers Ef728M [86],Tef1R [87], ef1 [88], ef2 [89], EF1-1018F and EF1-1620R [90] and the beta-tubulin gene was amplified using primers Bt2a and Bt2b [91]. Polymerase chain reactions (PCR) were carried out in 25-µL reaction volumes with 1 µL of genomic DNA (50 ng µL^−1^), 2.5 µL of 10× DreamTaq buffer (including 20 mM MgCl2; Thermo Fisher Scientific, Waltham, MA, USA), 0.2 µL of DreamTaq DNA polymerase (5 U µL^−1^; Thermo Fisher Scientific, Waltham, MA, USA), 0.2 µL of each primer (100 mmol L^−1^) and 2 µL of dNTP mix (2.5 mM; Sigma-Aldrich, Saint Louis, MO, USA), replenished with sterile distilled water to 25 µL, using a C1000 Thermal Cycler (Bio-Rad, Hercules, CA, USA). The following thermal conditions were applied for the reactions: initial denaturation at 94 °C for 5 min; 35 cycles of denaturation at 94 °C for 45 s, annealing at 52 °C for the ITS (internal transcribed spacer), LSU (large subunit) and SSU (small subunit) regions (or 55 °C for 45 s for the βtub gene fragment; for the tef1 gene fragment, this depended on the primer used: 53 °C for primers ef1 and ef2, 63° C for Ef728M and Tef1R and 56 °C for 45 s for EF1-1018F and EF1-1620R), extension at 72 °C for 1 min; and a final extension at 72 °C for 10 min. The obtained PCR products were electrophoresed, visualized under UV light and photographed (Syngene UV visualizer). Visibly clear PCR products were purified with exonuclease I and shrimp alkaline phosphatase. The samples were incubated in a C1000 Thermal Cycler (Bio-Rad, Hercules, CA, USA) using the following temperature profiles: 37 °C for 15 min, then 85 °C for 15 min and finally 4 °C for 5 min. The sequencing reactions of ITS, SSU, LSU, *tef1* and *βtub* amplicons were performed using the ABI Prism BigDye Terminator Cycle Sequencing Ready Reaction Kit (Applied Biosystems, Life Technologies Corporation, Austin, USA) according to the manufacturer’s instructions. The cleaning of the sequencing products was performed on MultiScreen Filter Plates HTS (Merck Millipore, Burlington, MA, United States) and by using 0.4 g of Sephadex^®^ G-50 beads with a diameter of 20–50 µm (Sigma-Aldrich, Saint Louis, MO, United States). Sequencing reads were performed at the Sequencing Laboratory in the Institute of Biochemistry and Biophysics in Warsaw. The sequences were edited and assembled using Chromas software (version 1.43, 2004; Technelysium Pty Ltd., South Brisbane, QLD, Australia). For the identification of species, the sequences were subjected to BLASTn analysis (NCBI, http://blast.ncbi.nlm.nih.gov/). Fungal diversity associated with the rhizosphere and internal root tissues in two water regimes, wheat varieties and mycorrhizal inoculation levels was evaluated using Shannon’s biodiversity index (H) (6) and Shannon evenness (E) (7) index [92]. Indices were calculated according to the following equations:

(6) H=−[Pilnpi]

where *H* is the Shannon’s index, *pi* is the proportional abundance of the *i*th species (ni/N), where *p* is the proportion (n/N) of individuals of one particular species found (*n*) divided by the total number of individuals found (N), ln is the natural log and Σ is the sum of the calculations.

(7) $$E=\frac{H}{\ln S}$$

where *E* is the Shannon evenness index and *S* is the number of species.

### 4.4. Statistical Analysis

Statistica 12.0 (StatSoft Inc., Krakow, Poland) [93] and Microsoft Excel 2013 software packages were used for all statistical analyses. The effect of the experimental factor (water regime, wheat variety, mycorrhizal inoculation level) on the physiological state of the plants, biomass production and root colonization cover (%) was tested using three-way ANOVA with replicates. Based on the ANOVA output, Duncan’s multiple-range test was performed to make comparisons at a significance level of *p* = 0.05. The following statistical model (8) was used:

(8) $$y_{ijkl}=\mu +\alpha_{i}+\beta_{j}+\gamma_{k}+{\left(\beta \gamma \right)}_{jk}+{\left(\alpha \beta \right)}_{ij}+{\left(\alpha \gamma \right)}_{ik}+{\left(\alpha \beta \gamma \right)}_{ijk}+e_{ijkl}$$

where *y_ijk_* is the estimated value of variables in the presence or absence of drought stress (*i* = 1, 2), wheat variety (*j* = 1, 2…, 4) and mycorrhizal treatment (*k* = 1, 2, 3), µ is the overall average, $\alpha_{i}$ is the effect of the occurrence or absence of drought stress, $\beta_{j}$ is the effect of the wheat variety, $\gamma_{k}$ is the effect of mycorrhizal inoculation, with appropriate interactions of these factors and $e_{ijkl}$ is the random error. Percentage data were analyzed after arcsin transformation. A heat map was proposed as a graphical presentation of appropriately transformed data in regard to Shannon’s biodiversity index (H′) and Shannon evenness (E) index. Data transformation using ‘normalize’ was used to compare and group different data. In addition, heatmap cluster Figure 1 and Figure 2 were used to summarize the data from the experiment according to Euclidean distance [94]. The relationship between the parameters was determined with the Pearson correlation coefficient. Interpretation of Pearson’s linear correlation coefficient was conducted according to Stanisz [95].

## 5. Conclusions

The results observed in this study showed that common wheat and spelt varieties significantly differed in their response to drought and, hence, their drought tolerance, although it is difficult to clearly indicate which variety was the most tolerant. However, based on a comprehensive analysis (physiological and biometric measurements) our results suggest that the spelt variety ‘Badenstern’ may be the most drought tolerant, followed by ‘Zollernspelz’ and ‘Badenkrone,’ while the common wheat variety ‘Dakotana’ was the most drought sensitive. ‘Badenstern’ would appear to be a suitable variety for use in future breeding programs, to cross with modern wheat varieties in order to confer more drought resistance to the latter. ‘Badenstern’ was found to improve drought stress tolerance through photoprotective responses, such as improved photosynthetic and transpiration rates and WUE, which resulted in better growth performance both underground (length and volume of roots) and aboveground (whole plant and stalk) biomass. In addition, the spelt variety ‘Zollernspelz’ exhibited enhanced PSII efficiency and electron transport rate, and displayed greater plant and root dry weights under drought conditions. The degree of drought tolerance depends on the interactions between the wheat varieties and symbiosis with the microorganism. It has been shown that the inoculation of spelt wheat ‘Badenstern’ with the mycorrhizal fungi *G. irregulare* contributed to better growth performance as evidenced by increased whole plant and stalk dry matter accumulation, as well as enhanced root length and volume. Inoculation of ‘Zollernspelz’ with AMF enhanced the photochemical efficiency of PSII and significantly improved root growth under drought conditions, which was confirmed by greater above- and below-ground values. In conclusion, this study provided evidence that AMF have the potential to be beneficial for plant growth and for dry matter accumulation in spelt plants grown under water deficits. The beneficial activity of AMF in the common wheat variety ‘Dakotana’ was most likely to be expressed under drought rather than in well-watered conditions. Our research leads to the conclusion that AMF must tightly fit with the plant species or even variety, to ensure maximum benefits from their association.

## Figures and Tables

**Table 1:** Three-way ANOVA showing the statistical significance of the effect of water regime and mycorrhizal inoculation on dry weight of aboveground part of plants (Ab), spike (Sp), stalk (Sk), roots (R), root volume (v), length (l), root colonization (rc), relative water content (RWC), quantum yield of photosystem II (Y), electron transport rate (ETR), maximum photochemical efficiency of PSII (Fv/Fm), photosynthetic rate (A), transpiration rate (E) and water use efficiency (WUE), in the common wheat variety ‘Dakotana’ and spelt wheat varieties ‘Badenstern,’ ‘Badenkrone’ and ‘Zollernspelz.’

Factors	df	Dry Weight of				Root		rc	RWC	Y	ETR	Fv/Fm	A	E	WUE
		Ab	Sp	Sk	R	v	l								
Water (W)	1	**	**	**	**	**	**	ns	**	**	**	**	**	**	ns
Variety (G)	3	**	**	**	**	**	**	**	**	ns	**	**	ns	**	**
Inoculation (I)	2	**	*	**	**	**	**	**	**	ns	ns	ns	*	ns	**
W × G	3	**	**	*	**	**	**	**	**	*	**	**	**	**	**
W × I	2	**	**	**	**	**	**	**	*	*	**	ns	**	**	*
G × I	6	**	**	**	**	**	**	**	**	**	**	**	**	**	**
W × G × I	6	**	**	**	**	**	**	**	**	*	**	**	**	**	ns
Error	120	-	-	-	-	-	-	-	-	-	-	-	--	-	-

ns: non-significant; **: significance three-way ANOVA at *p* < 0.01; * significance three-way ANOVA at *p* < 0.05.

**Table 2:** Shannon’s biodiversity (H) and Shannon evenness (E) indices for wheat variety.

Variety	‘Dakotana’	‘Badenstern’	‘Badenkrone’	‘Zollernspelz’
H	2.52	2.49	2.42	2.58
E	0.87	0.88	0.94	0.93

**Table 3:** Shannon’s biodiversity (H) and Shannon evenness (E) indices for water regime and mycorrhizal inoculation (i—with *G. irregulare* inoculation, c—control and ni—natural inoculation).

Water Regime	Mycorrhizal Inoculation	H	E
Well-watered	c	2.47	0.91
	ni	2.54	0.94
	i	2.13	0.86
Drought	c	2.10	0.97
	ni	2.11	0.92
	i	1.41	0.79

**Table 4:** Shannon’s biodiversity (H) and Shannon evenness (E) indices for water regime, wheat variety and mycorrhizal inoculation interaction (i—with *G. irregulare* inoculation, c—control and ni—natural inoculation).

Variety	‘Dakotana’			‘Badenstern’			‘Badenkrone’			‘Zollernspelz’		
	i	c	ni	i	c	ni	i	c	ni	i	c	ni
Well-watered												
H	1.04	1.15	2.04	1.55	1.79	1.95	1.56	1.39	0.69	1.04	1.61	1.61
E	0.95	0.83	0.98	0.96	1.00	1.00	0.97	1.00	1.00	0.95	1.00	1.00
Drought												
H	1.05	0.56	1.56	0.64	1.10	0.64	0.64	1.33	1.39	1.33	1.33	1.39
E	0.76	0.81	0.97	0.92	1.00	0.92	0.92	0.96	1.00	0.96	0.96	1.00

## Supplementary Materials

The following are available online at https://www.mdpi.com/1422-0067/21/21/7987/s1, Table S1: Correlation coefficients between agronomic traits and physiological parameters in mycorrhizal inoculation (i) with *G. irregulare* under well-watered and drought conditions, Table S2: Correlation coefficients between agronomic traits and physiological parameters in plants grown in sterile soil under well-watered and drought conditions, Table S3: Correlation coefficients between agronomic traits and physiological parameters in plants with natural inoculation under well-watered and drought conditions, Table S4: Correlation coefficients between agronomic traits and physiological parameters in the common wheat variety ‘Dakotana’ under two water regimes (well-watered, drought), Table S5: Correlation coefficients between agronomic traits and physiological parameters in the spelt wheat variety ‘Badenstern’ under two water regimes (well-watered, drought), Table S6: Correlation coefficients between agronomic traits and physiological parameters in the spelt wheat variety ‘Badenkrone’ under two water regimes (well-watered, drought), Table S7: Correlation coefficients between agronomic traits and physiological parameters in the spelt wheat variety ‘Zollernspelz’ (Z) under two water regimes (well-watered, drought).
