# Supplementary material for: Growth and Photosynthetic Activity of Selected Spelt Varieties (Triticum aestivum ssp. spelta L.) Cultivated under Drought Conditions with Different Endophytic Core Microbiomes
Source: Int J Mol Sci. 2020 Oct 27;21(21):7987. doi: 10.3390/ijms21217987 (PMC7662716; doi:10.3390/ijms21217987)
Supplement: Supplementary file 1 [file ijms-21-07987-s001.pdf]

**Table S1**: Correlation coefficients between agronomic traits and physiological parameters in mycorrhizal inoculation (i) with *G. irregulare* under well-watered and drought conditions.

| Traits | R | l | v | Ab | Sp | Sk | rc | Fv/Fm | Y | ETR | E | A | WUE | RWC |
|---|---|---|---|---|---|---|---|---|---|---|---|---|---|---|
| | Well-watered | | | | | | | | | | | | | |
| R | 1.00 | 0.74 | -0.63 | 0.74 | 0.56 | 0.77 | 0.42 | -0.60 | -0.27 | -0.65 | -0.26 | -0.49 | -0.24 | 0.43 |
| l | 0.74 | 1.00 | -0.69 | 0.33 | 0.32 | 0.31 | 0.63 | -0.34 | -0.18 | -0.28 | -0.29 | -0.63 | -0.43 | 0.41 |
| v | -0.63 | -0.69 | 1.00 | -0.75 | -0.83 | -0.52 | -0.96 | 0.06 | -0.39 | -0.04 | -0.24 | 0.16 | 0.34 | -0.86 |
| Ab | 0.74 | 0.33 | -0.75 | 1.00 | 0.93 | 0.89 | 0.58 | -0.30 | 0.15 | -0.30 | 0.26 | 0.08 | -0.05 | 0.70 |
| Sp | 0.56 | 0.32 | -0.83 | 0.93 | 1.00 | 0.71 | 0.75 | 0.03 | 0.38 | -0.06 | 0.50 | 0.17 | -0.17 | 0.82 |
| Sk | 0.77 | 0.31 | -0.52 | 0.89 | 0.71 | 1.00 | 0.31 | -0.52 | -0.03 | -0.49 | 0.00 | 0.07 | 0.14 | 0.38 |
| rc | 0.42 | 0.63 | -0.96 | 0.58 | 0.75 | 0.31 | 1.00 | 0.12 | 0.50 | 0.27 | 0.34 | -0.09 | -0.37 | 0.86 |
| Fv/Fm | -0.60 | -0.34 | 0.06 | -0.30 | 0.03 | -0.52 | 0.12 | 1.00 | 0.46 | 0.53 | 0.60 | 0.28 | -0.24 | 0.14 |
| Y | -0.27 | -0.18 | -0.39 | 0.15 | 0.38 | -0.03 | 0.50 | 0.46 | 1.00 | 0.68 | 0.55 | 0.42 | -0.04 | 0.43 |
| ETR | -0.65 | -0.28 | -0.04 | -0.30 | -0.06 | -0.49 | 0.27 | 0.53 | 0.68 | 1.00 | 0.49 | 0.41 | -0.06 | 0.16 |
| E | -0.26 | -0.29 | -0.24 | 0.26 | 0.50 | 0.00 | 0.34 | 0.60 | 0.55 | 0.49 | 1.00 | 0.45 | -0.35 | 0.58 |
| A | -0.49 | -0.63 | 0.16 | 0.08 | 0.17 | 0.07 | -0.09 | 0.28 | 0.42 | 0.41 | 0.45 | 1.00 | 0.67 | -0.11 |
| WUE | -0.24 | -0.43 | 0.34 | -0.05 | -0.17 | 0.14 | -0.37 | -0.24 | -0.04 | -0.06 | -0.35 | 0.67 | 1.00 | -0.56 |
| RWC | 0.43 | 0.41 | -0.86 | 0.70 | 0.82 | 0.38 | 0.86 | 0.14 | 0.43 | 0.16 | 0.58 | -0.11 | -0.56 | 1.00 |
| | drought | | | | | | | | | | | | | |
| R | 1.00 | 0.64 | -0.23 | -0.69 | -0.74 | -0.19 | -0.35 | -0.50 | -0.52 | -0.37 | -0.66 | -0.90 | -0.70 | -0.91 |
| l | 0.64 | 1.00 | 0.22 | -0.59 | -0.78 | 0.11 | 0.35 | -0.19 | -0.15 | -0.40 | 0.07 | -0.69 | -0.81 | -0.75 |
| v | -0.23 | 0.22 | 1.00 | -0.52 | -0.40 | -0.47 | 0.91 | 0.10 | 0.07 | -0.73 | 0.48 | -0.08 | -0.22 | 0.07 |
| Ab | -0.69 | -0.59 | -0.52 | 1.00 | 0.90 | 0.62 | -0.29 | 0.38 | 0.41 | 0.87 | 0.32 | 0.81 | 0.69 | 0.69 |
| Sp | -0.74 | -0.78 | -0.40 | 0.90 | 1.00 | 0.43 | -0.28 | 0.39 | 0.46 | 0.72 | 0.27 | 0.83 | 0.73 | 0.74 |
| Sk | -0.19 | 0.11 | -0.47 | 0.62 | 0.43 | 1.00 | -0.13 | 0.28 | 0.29 | 0.73 | 0.33 | 0.26 | 0.03 | 0.11 |
| rc | -0.35 | 0.35 | 0.91 | -0.29 | -0.28 | -0.13 | 1.00 | 0.29 | 0.27 | -0.49 | 0.71 | 0.06 | -0.21 | 0.13 |
| Fv/Fm | -0.50 | -0.19 | 0.10 | 0.38 | 0.39 | 0.28 | 0.29 | 1.00 | 0.59 | 0.34 | 0.29 | 0.48 | 0.38 | 0.32 |
| Y | -0.52 | -0.15 | 0.07 | 0.41 | 0.46 | 0.29 | 0.27 | 0.59 | 1.00 | 0.28 | 0.50 | 0.59 | 0.33 | 0.50 |
| ETR | -0.37 | -0.40 | -0.73 | 0.87 | 0.72 | 0.73 | -0.49 | 0.34 | 0.28 | 1.00 | 0.07 | 0.59 | 0.53 | 0.43 |
| E | -0.66 | 0.07 | 0.48 | 0.32 | 0.27 | 0.33 | 0.71 | 0.29 | 0.50 | 0.07 | 1.00 | 0.43 | -0.02 | 0.43 |
| A | -0.90 | -0.69 | -0.08 | 0.81 | 0.83 | 0.26 | 0.06 | 0.48 | 0.59 | 0.59 | 0.43 | 1.00 | 0.87 | 0.94 |
| WUE | -0.70 | -0.81 | -0.22 | 0.69 | 0.73 | 0.03 | -0.21 | 0.38 | 0.33 | 0.53 | -0.02 | 0.87 | 1.00 | 0.86 |
| RWC | -0.91 | -0.75 | 0.07 | 0.69 | 0.74 | 0.11 | 0.13 | 0.32 | 0.50 | 0.43 | 0.43 | 0.94 | 0.86 | 1.00 |

**Table S2**: Correlation coefficients between agronomic traits and physiological parameters in plants grown in sterile soil under well-watered and drought conditions.

| Traits | R | l | v | Ab | Sp | Sk | rc | Fv/Fm | Y | ETR | E | A | WUE | RWC |
|---|---|---|---|---|---|---|---|---|---|---|---|---|---|---|
| | Well-watered | | | | | | | | | | | | | |
| R | 1.00 | 0.83 | -0.45 | -0.53 | -0.65 | -0.13 | -0.86 | -0.45 | -0.25 | 0.91 | -0.60 | -0.62 | -0.11 | -0.22 |
| l | 0.83 | 1.00 | -0.54 | -0.28 | -0.53 | 0.10 | -0.92 | -0.15 | 0.04 | 0.90 | -0.74 | -0.74 | -0.21 | 0.11 |
| v | -0.45 | -0.54 | 1.00 | -0.41 | 0.36 | -0.74 | 0.26 | -0.35 | -0.69 | -0.46 | 0.16 | 0.93 | 0.78 | -0.47 |
| Ab | -0.53 | -0.28 | -0.41 | 1.00 | 0.52 | 0.79 | 0.53 | 0.62 | 0.71 | -0.45 | 0.40 | -0.19 | -0.53 | 0.66 |
| Sp | -0.65 | -0.53 | 0.36 | 0.52 | 1.00 | -0.11 | 0.53 | 0.05 | 0.00 | -0.50 | 0.65 | 0.44 | -0.13 | 0.35 |
| Sk | -0.13 | 0.10 | -0.74 | 0.79 | -0.11 | 1.00 | 0.20 | 0.69 | 0.81 | -0.13 | -0.02 | -0.54 | -0.52 | 0.54 |
| rc | -0.86 | -0.92 | 0.26 | 0.53 | 0.53 | 0.20 | 1.00 | 0.34 | 0.25 | -0.84 | 0.77 | 0.50 | -0.05 | 0.14 |
| Fv/Fm | -0.45 | -0.15 | -0.35 | 0.62 | 0.05 | 0.69 | 0.34 | 1.00 | 0.77 | -0.34 | 0.18 | -0.15 | -0.32 | 0.49 |
| Y | -0.25 | 0.04 | -0.69 | 0.71 | 0.00 | 0.81 | 0.25 | 0.77 | 1.00 | -0.15 | 0.21 | -0.53 | -0.71 | 0.70 |

| | | | | | | | | | | | | | | |
|---|---|---|---|---|---|---|---|---|---|---|---|---|---|---|
| ETR | 0.91 | 0.90 | -0.46 | -0.45 | -0.50 | -0.13 | -0.84 | -0.34 | -0.15 | 1.00 | -0.57 | -0.67 | -0.24 | -0.03 |
| E | -0.60 | -0.74 | 0.16 | 0.40 | 0.65 | -0.02 | 0.77 | 0.18 | 0.21 | -0.57 | 1.00 | 0.39 | -0.35 | 0.39 |
| A | -0.62 | -0.74 | 0.93 | -0.19 | 0.44 | -0.54 | 0.50 | -0.15 | -0.53 | -0.67 | 0.39 | 1.00 | 0.71 | -0.34 |
| WUE | -0.11 | -0.21 | 0.78 | -0.53 | -0.13 | -0.52 | -0.05 | -0.32 | -0.71 | -0.24 | -0.35 | 0.71 | 1.00 | -0.73 |
| RWC | -0.22 | 0.11 | -0.47 | 0.66 | 0.35 | 0.54 | 0.14 | 0.49 | 0.70 | -0.03 | 0.39 | -0.34 | -0.73 | 1.00 |
| | drought | | | | | | | | | | | | | |
| R | 1.00 | 0.92 | 0.08 | -0.88 | -0.88 | -0.53 | -0.84 | -0.44 | -0.57 | -0.88 | 0.75 | -0.56 | -0.83 | -0.58 |
| l | 0.92 | 1.00 | 0.34 | -0.81 | -0.80 | -0.52 | -0.78 | -0.04 | -0.39 | -0.72 | 0.66 | -0.44 | -0.72 | -0.22 |
| v | 0.08 | 0.34 | 1.00 | -0.44 | -0.27 | -0.69 | 0.31 | 0.58 | 0.25 | 0.16 | 0.45 | 0.23 | -0.16 | 0.57 |
| Ab | -0.88 | -0.81 | -0.44 | 1.00 | 0.93 | 0.85 | 0.49 | 0.34 | 0.46 | 0.71 | -0.91 | 0.41 | 0.80 | 0.41 |
| Sp | -0.88 | -0.80 | -0.27 | 0.93 | 1.00 | 0.67 | 0.60 | 0.36 | 0.50 | 0.72 | -0.74 | 0.57 | 0.78 | 0.49 |
| Sk | -0.53 | -0.52 | -0.69 | 0.85 | 0.67 | 1.00 | 0.05 | 0.11 | 0.25 | 0.33 | -0.84 | 0.15 | 0.57 | 0.10 |
| rc | -0.84 | -0.78 | 0.31 | 0.49 | 0.60 | 0.05 | 1.00 | 0.39 | 0.55 | 0.82 | -0.32 | 0.61 | 0.60 | 0.57 |
| Fv/Fm | -0.44 | -0.04 | 0.58 | 0.34 | 0.36 | 0.11 | 0.39 | 1.00 | 0.54 | 0.62 | -0.39 | 0.36 | 0.43 | 0.92 |
| Y | -0.57 | -0.39 | 0.25 | 0.46 | 0.50 | 0.25 | 0.55 | 0.54 | 1.00 | 0.57 | -0.27 | 0.22 | 0.35 | 0.52 |
| ETR | -0.88 | -0.72 | 0.16 | 0.71 | 0.72 | 0.33 | 0.82 | 0.62 | 0.57 | 1.00 | -0.60 | 0.47 | 0.61 | 0.69 |
| E | 0.75 | 0.66 | 0.45 | -0.91 | -0.74 | -0.84 | -0.32 | -0.39 | -0.27 | -0.60 | 1.00 | -0.30 | -0.81 | -0.39 |
| A | -0.56 | -0.44 | 0.23 | 0.41 | 0.57 | 0.15 | 0.61 | 0.36 | 0.22 | 0.47 | -0.30 | 1.00 | 0.62 | 0.66 |
| WUE | -0.83 | -0.72 | -0.16 | 0.80 | 0.78 | 0.57 | 0.60 | 0.43 | 0.35 | 0.61 | -0.81 | 0.62 | 1.00 | 0.53 |
| RWC | -0.58 | -0.22 | 0.57 | 0.41 | 0.49 | 0.10 | 0.57 | 0.92 | 0.52 | 0.69 | -0.39 | 0.66 | 0.53 | 1.00 |

**Table S3**: Correlation coefficients between agronomic traits and physiological parameters in plants with natural inoculation under well-watered and drought conditions.

| Traits | R | l | v | Ab | Sp | Sk | rc | Fv/Fm | Y | ETR | E | A | WUE | RWC |
|---|---|---|---|---|---|---|---|---|---|---|---|---|---|---|
| | Well-watered | | | | | | | | | | | | | |
| R | 1.00 | -0.37 | -0.14 | -0.37 | -0.22 | -0.58 | 0.12 | -0.91 | -0.58 | -0.92 | -0.42 | -0.47 | 0.55 | -0.43 |
| l | -0.37 | 1.00 | -0.25 | -0.36 | -0.49 | -0.18 | 0.19 | 0.24 | 0.02 | 0.06 | -0.25 | 0.21 | 0.23 | 0.52 |
| v | -0.14 | -0.25 | 1.00 | -0.39 | -0.37 | -0.37 | -0.99 | 0.07 | -0.51 | 0.23 | -0.36 | -0.62 | -0.17 | -0.10 |
| Ab | -0.37 | -0.36 | -0.39 | 1.00 | 0.98 | 0.92 | 0.46 | 0.48 | 0.83 | 0.49 | 0.94 | 0.69 | -0.54 | -0.13 |
| Sp | -0.22 | -0.49 | -0.37 | 0.98 | 1.00 | 0.83 | 0.44 | 0.32 | 0.72 | 0.39 | 0.91 | 0.56 | -0.53 | -0.20 |
| Sk | -0.58 | -0.18 | -0.37 | 0.92 | 0.83 | 1.00 | 0.43 | 0.68 | 0.88 | 0.61 | 0.85 | 0.79 | -0.50 | 0.01 |
| rc | 0.12 | 0.19 | -0.99 | 0.46 | 0.44 | 0.43 | 1.00 | -0.03 | 0.55 | -0.20 | 0.42 | 0.67 | 0.17 | 0.09 |
| Fv/Fm | -0.91 | 0.24 | 0.07 | 0.48 | 0.32 | 0.68 | -0.03 | 1.00 | 0.61 | 0.84 | 0.52 | 0.64 | -0.49 | 0.14 |
| Y | -0.58 | 0.02 | -0.51 | 0.83 | 0.72 | 0.88 | 0.55 | 0.61 | 1.00 | 0.56 | 0.86 | 0.85 | -0.57 | 0.10 |
| ETR | -0.92 | 0.06 | 0.23 | 0.49 | 0.39 | 0.61 | -0.20 | 0.84 | 0.56 | 1.00 | 0.53 | 0.35 | -0.74 | 0.34 |
| E | -0.42 | -0.25 | -0.36 | 0.94 | 0.91 | 0.85 | 0.42 | 0.52 | 0.86 | 0.53 | 1.00 | 0.71 | -0.70 | -0.12 |
| A | -0.47 | 0.21 | -0.62 | 0.69 | 0.56 | 0.79 | 0.67 | 0.64 | 0.85 | 0.35 | 0.71 | 1.00 | -0.23 | 0.07 |
| WUE | 0.55 | 0.23 | -0.17 | -0.54 | -0.53 | -0.50 | 0.17 | -0.49 | -0.57 | -0.74 | -0.70 | -0.23 | 1.00 | 0.02 |
| RWC | -0.43 | 0.52 | -0.10 | -0.13 | -0.20 | 0.01 | 0.09 | 0.14 | 0.10 | 0.34 | -0.12 | 0.07 | 0.02 | 1.00 |
| | drought | | | | | | | | | | | | | |
| R | 1.00 | -0.15 | -0.26 | -0.47 | -0.48 | -0.44 | 0.57 | -0.77 | -0.26 | -0.09 | 0.29 | 0.15 | -0.13 | -0.23 |
| l | -0.15 | 1.00 | 0.40 | -0.70 | -0.65 | -0.72 | 0.71 | -0.28 | -0.85 | -0.91 | -0.33 | -0.83 | -0.65 | -0.84 |
| v | -0.26 | 0.40 | 1.00 | -0.32 | -0.30 | -0.32 | 0.12 | 0.15 | -0.38 | -0.27 | -0.20 | -0.24 | -0.12 | -0.44 |
| Ab | -0.47 | -0.70 | -0.32 | 1.00 | 0.98 | 0.94 | -0.91 | 0.69 | 0.80 | 0.74 | 0.03 | 0.59 | 0.68 | 0.89 |
| Sp | -0.48 | -0.65 | -0.30 | 0.98 | 1.00 | 0.88 | -0.89 | 0.74 | 0.77 | 0.71 | -0.05 | 0.59 | 0.73 | 0.87 |
| Sk | -0.44 | -0.72 | -0.32 | 0.94 | 0.88 | 1.00 | -0.90 | 0.58 | 0.84 | 0.80 | 0.02 | 0.49 | 0.63 | 0.83 |
| rc | 0.57 | 0.71 | 0.12 | -0.91 | -0.89 | -0.90 | 1.00 | -0.79 | -0.87 | -0.82 | -0.04 | -0.57 | -0.64 | -0.84 |

| | | | | | | | | | | | | | | |
|---|---|---|---|---|---|---|---|---|---|---|---|---|---|---|
| Fv/Fm | -0.77 | -0.28 | 0.15 | 0.69 | 0.74 | 0.58 | -0.79 | 1.00 | 0.52 | 0.43 | -0.19 | 0.40 | 0.60 | 0.58 |
| Y | -0.26 | -0.85 | -0.38 | 0.80 | 0.77 | 0.84 | -0.87 | 0.52 | 1.00 | 0.90 | 0.09 | 0.63 | 0.65 | 0.84 |
| ETR | -0.09 | -0.91 | -0.27 | 0.74 | 0.71 | 0.80 | -0.82 | 0.43 | 0.90 | 1.00 | -0.03 | 0.64 | 0.76 | 0.85 |
| E | 0.29 | -0.33 | -0.20 | 0.03 | -0.05 | 0.02 | -0.04 | -0.19 | 0.09 | -0.03 | 1.00 | 0.47 | -0.33 | 0.13 |
| A | 0.15 | -0.83 | -0.24 | 0.59 | 0.59 | 0.49 | -0.57 | 0.40 | 0.63 | 0.64 | 0.47 | 1.00 | 0.66 | 0.76 |
| WUE | -0.13 | -0.65 | -0.12 | 0.68 | 0.73 | 0.63 | -0.64 | 0.60 | 0.65 | 0.76 | -0.33 | 0.66 | 1.00 | 0.74 |
| RWC | -0.23 | -0.84 | -0.44 | 0.89 | 0.87 | 0.83 | -0.84 | 0.58 | 0.84 | 0.85 | 0.13 | 0.76 | 0.74 | 1.00 |

**Table S4**: Correlation coefficients between agronomic traits and physiological parameters in the common wheat variety ‘Dakotana’ under two water regimes (well-watered, drought).

| Traits | R | l | v | Ab | Sp | Sk | rc | Fv/Fm | Y | ETR | E | A | WUE | RWC |
|---|---|---|---|---|---|---|---|---|---|---|---|---|---|---|
| | Well-watered | | | | | | | | | | | | | |
| R | 1.00 | 0.21 | 0.54 | 0.92 | 0.81 | 0.90 | -0.55 | 0.11 | 0.30 | -0.10 | -0.29 | -0.79 | -0.50 | 0.46 |
| l | 0.21 | 1.00 | -0.70 | 0.53 | 0.55 | 0.45 | 0.70 | -0.01 | -0.23 | -0.01 | -0.29 | -0.25 | 0.22 | 0.93 |
| v | 0.54 | -0.70 | 1.00 | 0.21 | 0.10 | 0.27 | -1.00 | 0.03 | 0.38 | -0.06 | 0.08 | -0.34 | -0.56 | -0.46 |
| Ab | 0.92 | 0.53 | 0.21 | 1.00 | 0.91 | 0.96 | -0.22 | 0.05 | 0.10 | -0.05 | -0.38 | -0.80 | -0.36 | 0.72 |
| Sp | 0.81 | 0.55 | 0.10 | 0.91 | 1.00 | 0.75 | -0.11 | 0.05 | -0.15 | -0.17 | -0.42 | -0.88 | -0.37 | 0.62 |
| Sk | 0.90 | 0.45 | 0.27 | 0.96 | 0.75 | 1.00 | -0.27 | 0.08 | 0.22 | 0.02 | -0.31 | -0.64 | -0.30 | 0.71 |
| rc | -0.55 | 0.70 | -1.00 | -0.22 | -0.11 | -0.27 | 1.00 | -0.05 | -0.40 | 0.06 | -0.10 | 0.35 | 0.59 | 0.45 |
| Fv/Fm | 0.11 | -0.01 | 0.03 | 0.05 | 0.05 | 0.08 | -0.05 | 1.00 | -0.09 | -0.50 | -0.08 | 0.23 | 0.16 | 0.07 |
| Y | 0.30 | -0.23 | 0.38 | 0.10 | -0.15 | 0.22 | -0.40 | -0.09 | 1.00 | 0.44 | 0.11 | -0.10 | -0.26 | -0.01 |
| ETR | -0.10 | -0.01 | -0.06 | -0.05 | -0.17 | 0.02 | 0.06 | -0.50 | 0.44 | 1.00 | 0.36 | -0.06 | -0.32 | 0.06 |
| E | -0.29 | -0.29 | 0.08 | -0.38 | -0.42 | -0.31 | -0.10 | -0.08 | 0.11 | 0.36 | 1.00 | 0.33 | -0.53 | -0.32 |
| A | -0.79 | -0.25 | -0.34 | -0.80 | -0.88 | -0.64 | 0.35 | 0.23 | -0.10 | -0.06 | 0.33 | 1.00 | 0.59 | -0.33 |
| WUE | -0.50 | 0.22 | -0.56 | -0.36 | -0.37 | -0.30 | 0.59 | 0.16 | -0.26 | -0.32 | -0.53 | 0.59 | 1.00 | 0.12 |
| RWC | 0.46 | 0.93 | -0.46 | 0.72 | 0.62 | 0.71 | 0.45 | 0.07 | -0.01 | 0.06 | -0.32 | -0.33 | 0.12 | 1.00 |
| | drought | | | | | | | | | | | | | |
| R | 1.00 | -0.28 | 0.19 | -0.57 | -0.07 | -0.07 | 0.94 | 0.31 | 0.38 | -0.17 | -0.76 | -0.96 | 0.22 | 0.28 |
| l | -0.28 | 1.00 | 0.19 | 0.44 | 0.35 | 0.07 | -0.19 | 0.59 | 0.04 | 0.12 | -0.20 | 0.41 | 0.64 | -0.19 |
| v | 0.19 | 0.19 | 1.00 | 0.66 | 0.59 | 0.06 | 0.49 | 0.16 | -0.13 | -0.26 | 0.06 | 0.07 | -0.18 | -0.85 |
| Ab | -0.57 | 0.44 | 0.66 | 1.00 | 0.62 | 0.08 | -0.27 | -0.01 | -0.33 | -0.05 | 0.53 | 0.75 | -0.27 | -0.88 |
| Sp | -0.07 | 0.35 | 0.59 | 0.62 | 1.00 | 0.50 | 0.20 | 0.62 | 0.27 | 0.16 | 0.11 | 0.22 | -0.06 | -0.62 |
| Sk | -0.07 | 0.07 | 0.06 | 0.08 | 0.50 | 1.00 | 0.00 | 0.38 | 0.68 | 0.40 | 0.22 | 0.08 | -0.19 | -0.18 |
| rc | 0.94 | -0.19 | 0.49 | -0.27 | 0.20 | 0.00 | 1.00 | 0.36 | 0.29 | -0.23 | -0.65 | -0.83 | 0.14 | -0.03 |
| Fv/Fm | 0.31 | 0.59 | 0.16 | -0.01 | 0.62 | 0.38 | 0.36 | 1.00 | 0.51 | 0.26 | -0.55 | -0.23 | 0.65 | 0.06 |
| Y | 0.38 | 0.04 | -0.13 | -0.33 | 0.27 | 0.68 | 0.29 | 0.51 | 1.00 | 0.59 | -0.34 | -0.41 | 0.04 | 0.26 |
| ETR | -0.17 | 0.12 | -0.26 | -0.05 | 0.16 | 0.40 | -0.23 | 0.26 | 0.59 | 1.00 | -0.15 | 0.08 | 0.13 | 0.28 |
| E | -0.76 | -0.20 | 0.06 | 0.53 | 0.11 | 0.22 | -0.65 | -0.55 | -0.34 | -0.15 | 1.00 | 0.76 | -0.73 | -0.54 |
| A | -0.96 | 0.41 | 0.07 | 0.75 | 0.22 | 0.08 | -0.83 | -0.23 | -0.41 | 0.08 | 0.76 | 1.00 | -0.21 | -0.50 |
| WUE | 0.22 | 0.64 | -0.18 | -0.27 | -0.06 | -0.19 | 0.14 | 0.65 | 0.04 | 0.13 | -0.73 | -0.21 | 1.00 | 0.46 |
| RWC | 0.28 | -0.19 | -0.85 | -0.88 | -0.62 | -0.18 | -0.03 | 0.06 | 0.26 | 0.28 | -0.54 | -0.50 | 0.46 | 1.00 |

**Table S5**: Correlation coefficients between agronomic traits and physiological parameters in the spelt wheat variety ‘Badenstern’ under two water regimes (well-watered, drought).

| Traits | R | l | v | Ab | Sp | Sk | rc | Fv/Fm | Y | ETR | E | A | WUE | RWC |
|---|---|---|---|---|---|---|---|---|---|---|---|---|---|---|
| | Well-watered | | | | | | | | | | | | | |
| R | 1.00 | 0.19 | 0.99 | 0.92 | 0.37 | 0.96 | -0.98 | 0.17 | -0.66 | 0.73 | 0.09 | 0.50 | -0.19 | -0.22 |
| l | 0.19 | 1.00 | 0.08 | -0.02 | -0.46 | 0.19 | -0.23 | 0.39 | 0.19 | 0.33 | 0.28 | 0.25 | -0.21 | -0.14 |

| | | | | | | | | | | | | | | |
|---|---|---|---|---|---|---|---|---|---|---|---|---|---|---|
| v | 0.99 | 0.08 | 1.00 | 0.96 | 0.50 | 0.95 | -0.94 | 0.03 | -0.76 | 0.63 | -0.05 | 0.51 | -0.07 | -0.14 |
| Ab | 0.92 | -0.02 | 0.96 | 1.00 | 0.64 | 0.94 | -0.84 | -0.10 | -0.86 | 0.46 | -0.28 | 0.57 | 0.18 | -0.11 |
| Sp | 0.37 | -0.46 | 0.50 | 0.64 | 1.00 | 0.33 | -0.21 | -0.78 | -0.90 | -0.30 | -0.76 | 0.45 | 0.57 | 0.18 |
| Sk | 0.96 | 0.19 | 0.95 | 0.94 | 0.33 | 1.00 | -0.94 | 0.22 | -0.66 | 0.69 | 0.00 | 0.49 | -0.03 | -0.21 |
| rc | -0.98 | -0.23 | -0.94 | -0.84 | -0.21 | -0.94 | 1.00 | -0.32 | 0.51 | -0.84 | -0.25 | -0.40 | 0.33 | 0.25 |
| Fv/Fm | 0.17 | 0.39 | 0.03 | -0.10 | -0.78 | 0.22 | -0.32 | 1.00 | 0.50 | 0.69 | 0.78 | -0.07 | -0.58 | -0.57 |
| Y | -0.66 | 0.19 | -0.76 | -0.86 | -0.90 | -0.66 | 0.51 | 0.50 | 1.00 | 0.02 | 0.64 | -0.64 | -0.51 | -0.02 |
| ETR | 0.73 | 0.33 | 0.63 | 0.46 | -0.30 | 0.69 | -0.84 | 0.69 | 0.02 | 1.00 | 0.68 | 0.04 | -0.70 | -0.31 |
| E | 0.09 | 0.28 | -0.05 | -0.28 | -0.76 | 0.00 | -0.25 | 0.78 | 0.64 | 0.68 | 1.00 | -0.37 | -0.94 | -0.26 |
| A | 0.50 | 0.25 | 0.51 | 0.57 | 0.45 | 0.49 | -0.40 | -0.07 | -0.64 | 0.04 | -0.37 | 1.00 | 0.43 | -0.62 |
| WUE | -0.19 | -0.21 | -0.07 | 0.18 | 0.57 | -0.03 | 0.33 | -0.58 | -0.51 | -0.70 | -0.94 | 0.43 | 1.00 | 0.10 |
| RWC | -0.22 | -0.14 | -0.14 | -0.11 | 0.18 | -0.21 | 0.25 | -0.57 | -0.02 | -0.31 | -0.26 | -0.62 | 0.10 | 1.00 |
| | drought | | | | | | | | | | | | | |
| R | 1.00 | 0.00 | 0.35 | 0.23 | 0.19 | 0.35 | -0.96 | 0.07 | 0.80 | 0.78 | 0.64 | 0.38 | -0.25 | 0.80 |
| l | 0.00 | 1.00 | -0.84 | 0.86 | 0.17 | 0.73 | -0.19 | -0.05 | -0.22 | 0.50 | -0.39 | -0.74 | -0.47 | -0.29 |
| v | 0.35 | -0.84 | 1.00 | -0.83 | -0.36 | -0.62 | -0.10 | 0.07 | 0.55 | -0.18 | 0.58 | 0.58 | 0.10 | 0.65 |
| Ab | 0.23 | 0.86 | -0.83 | 1.00 | 0.47 | 0.88 | -0.46 | -0.01 | -0.11 | 0.65 | -0.23 | -0.36 | -0.24 | -0.21 |
| Sp | 0.19 | 0.17 | -0.36 | 0.47 | 1.00 | 0.19 | -0.26 | -0.49 | -0.09 | 0.24 | 0.33 | 0.14 | -0.16 | -0.18 |
| Sk | 0.35 | 0.73 | -0.62 | 0.88 | 0.19 | 1.00 | -0.54 | 0.03 | 0.10 | 0.62 | -0.13 | -0.25 | -0.23 | -0.06 |
| rc | -0.96 | -0.19 | -0.10 | -0.46 | -0.26 | -0.54 | 1.00 | -0.14 | -0.64 | -0.89 | -0.50 | -0.29 | 0.20 | -0.65 |
| Fv/Fm | 0.07 | -0.05 | 0.07 | -0.01 | -0.49 | 0.03 | -0.14 | 1.00 | -0.08 | 0.30 | -0.46 | 0.33 | 0.64 | 0.17 |
| Y | 0.80 | -0.22 | 0.55 | -0.11 | -0.09 | 0.10 | -0.64 | -0.08 | 1.00 | 0.33 | 0.57 | 0.27 | -0.28 | 0.93 |
| ETR | 0.78 | 0.50 | -0.18 | 0.65 | 0.24 | 0.62 | -0.89 | 0.30 | 0.33 | 1.00 | 0.19 | 0.05 | -0.23 | 0.41 |
| E | 0.64 | -0.39 | 0.58 | -0.23 | 0.33 | -0.13 | -0.50 | -0.46 | 0.57 | 0.19 | 1.00 | 0.37 | -0.41 | 0.56 |
| A | 0.38 | -0.74 | 0.58 | -0.36 | 0.14 | -0.25 | -0.29 | 0.33 | 0.27 | 0.05 | 0.37 | 1.00 | 0.62 | 0.36 |
| WUE | -0.25 | -0.47 | 0.10 | -0.24 | -0.16 | -0.23 | 0.20 | 0.64 | -0.28 | -0.23 | -0.41 | 0.62 | 1.00 | -0.18 |
| RWC | 0.80 | -0.29 | 0.65 | -0.21 | -0.18 | -0.06 | -0.65 | 0.17 | 0.93 | 0.41 | 0.56 | 0.36 | -0.18 | 1.00 |

**Table S6**: Correlation coefficients between agronomic traits and physiological parameters in the spelt wheat variety 'Badenkrone' under two water regimes (well-watered, drought).

| Traits | R | l | v | Ab | Sp | Sk | rc | Fv/Fm | Y | ETR | E | A | WUE | RWC |
|---|---|---|---|---|---|---|---|---|---|---|---|---|---|---|
| | Well-watered | | | | | | | | | | | | | |
| R | 1.00 | -0.10 | -0.96 | -0.60 | -0.45 | -0.05 | -0.24 | 0.02 | 0.08 | -0.16 | -0.91 | -0.39 | 0.15 | 0.68 |
| l | -0.10 | 1.00 | 0.16 | 0.34 | 0.36 | -0.02 | 0.32 | -0.53 | -0.20 | 0.19 | 0.20 | 0.41 | 0.44 | -0.16 |
| v | -0.96 | 0.16 | 1.00 | 0.80 | 0.68 | 0.06 | 0.51 | 0.05 | 0.04 | 0.11 | 0.98 | 0.61 | 0.11 | -0.74 |
| Ab | -0.60 | 0.34 | 0.80 | 1.00 | 0.98 | 0.14 | 0.91 | 0.11 | 0.21 | 0.06 | 0.83 | 0.91 | 0.63 | -0.66 |
| Sp | -0.45 | 0.36 | 0.68 | 0.98 | 1.00 | 0.16 | 0.95 | 0.05 | 0.21 | 0.06 | 0.71 | 0.89 | 0.72 | -0.56 |
| Sk | -0.05 | -0.02 | 0.06 | 0.14 | 0.16 | 1.00 | 0.11 | 0.19 | 0.30 | 0.11 | -0.02 | 0.31 | 0.40 | -0.47 |
| rc | -0.24 | 0.32 | 0.51 | 0.91 | 0.95 | 0.11 | 1.00 | 0.24 | 0.32 | 0.02 | 0.58 | 0.92 | 0.84 | -0.53 |
| Fv/Fm | 0.02 | -0.53 | 0.05 | 0.11 | 0.05 | 0.19 | 0.24 | 1.00 | 0.70 | -0.33 | 0.16 | 0.33 | 0.24 | -0.45 |
| Y | 0.08 | -0.20 | 0.04 | 0.21 | 0.21 | 0.30 | 0.32 | 0.70 | 1.00 | -0.78 | 0.17 | 0.41 | 0.41 | -0.34 |
| ETR | -0.16 | 0.19 | 0.11 | 0.06 | 0.06 | 0.11 | 0.02 | -0.33 | -0.78 | 1.00 | -0.01 | -0.01 | 0.01 | -0.16 |
| E | -0.91 | 0.20 | 0.98 | 0.83 | 0.71 | -0.02 | 0.58 | 0.16 | 0.17 | -0.01 | 1.00 | 0.69 | 0.18 | -0.76 |
| A | -0.39 | 0.41 | 0.61 | 0.91 | 0.89 | 0.31 | 0.92 | 0.33 | 0.41 | -0.01 | 0.69 | 1.00 | 0.83 | -0.75 |
| WUE | 0.15 | 0.44 | 0.11 | 0.63 | 0.72 | 0.40 | 0.84 | 0.24 | 0.41 | 0.01 | 0.18 | 0.83 | 1.00 | -0.41 |
| RWC | 0.68 | -0.16 | -0.74 | -0.66 | -0.56 | -0.47 | -0.53 | -0.45 | -0.34 | -0.16 | -0.76 | -0.75 | -0.41 | 1.00 |
| | drought | | | | | | | | | | | | | |

| | | | | | | | | | | | | | | |
|---|---|---|---|---|---|---|---|---|---|---|---|---|---|---|
| R | 1.00 | 0.60 | 0.99 | -0.78 | -0.87 | -0.60 | 0.64 | 0.25 | -0.49 | -0.86 | 0.83 | 0.03 | -0.89 | -0.02 |
| l | 0.60 | 1.00 | 0.71 | -0.06 | -0.51 | 0.21 | 0.97 | 0.02 | -0.28 | -0.83 | 0.93 | 0.47 | -0.74 | -0.54 |
| v | 0.99 | 0.71 | 1.00 | -0.70 | -0.86 | -0.50 | 0.73 | 0.22 | -0.48 | -0.92 | 0.90 | 0.10 | -0.92 | -0.12 |
| Ab | -0.78 | -0.06 | -0.70 | 1.00 | 0.83 | 0.94 | -0.14 | -0.40 | 0.31 | 0.46 | -0.39 | 0.12 | 0.58 | -0.33 |
| Sp | -0.87 | -0.51 | -0.86 | 0.83 | 1.00 | 0.60 | -0.58 | -0.38 | 0.36 | 0.70 | -0.76 | -0.15 | 0.80 | 0.02 |
| Sk | -0.60 | 0.21 | -0.50 | 0.94 | 0.60 | 1.00 | 0.14 | -0.32 | 0.20 | 0.24 | -0.10 | 0.22 | 0.34 | -0.49 |
| rc | 0.64 | 0.97 | 0.73 | -0.14 | -0.58 | 0.14 | 1.00 | 0.18 | -0.36 | -0.79 | 0.94 | 0.47 | -0.75 | -0.52 |
| Fv/Fm | 0.25 | 0.02 | 0.22 | -0.40 | -0.38 | -0.32 | 0.18 | 1.00 | -0.71 | -0.05 | 0.20 | -0.35 | -0.41 | -0.10 |
| Y | -0.49 | -0.28 | -0.48 | 0.31 | 0.36 | 0.20 | -0.36 | -0.71 | 1.00 | 0.41 | -0.43 | 0.61 | 0.64 | 0.38 |
| ETR | -0.86 | -0.83 | -0.92 | 0.46 | 0.70 | 0.24 | -0.79 | -0.05 | 0.41 | 1.00 | -0.91 | -0.17 | 0.88 | 0.29 |
| E | 0.83 | 0.93 | 0.90 | -0.39 | -0.76 | -0.10 | 0.94 | 0.20 | -0.43 | -0.91 | 1.00 | 0.31 | -0.90 | -0.41 |
| A | 0.03 | 0.47 | 0.10 | 0.12 | -0.15 | 0.22 | 0.47 | -0.35 | 0.61 | -0.17 | 0.31 | 1.00 | 0.05 | -0.01 |
| WUE | -0.89 | -0.74 | -0.92 | 0.58 | 0.80 | 0.34 | -0.75 | -0.41 | 0.64 | 0.88 | -0.90 | 0.05 | 1.00 | 0.32 |
| RWC | -0.02 | -0.54 | -0.12 | -0.33 | 0.02 | -0.49 | -0.52 | -0.10 | 0.38 | 0.29 | -0.41 | -0.01 | 0.32 | 1.00 |

**Table S7**: Correlation coefficients between agronomic traits and physiological parameters in the spelt wheat variety 'Zollernspelz' (Z) under two water regimes (well-watered, drought).

| Traits | R | l | v | Ab | Sp | Sk | rc | Fv/Fm | Y | ETR | E | A | WUE | RWC |
|---|---|---|---|---|---|---|---|---|---|---|---|---|---|---|
| | Well-watered | | | | | | | | | | | | | |
| R | 1.00 | 0.60 | -0.77 | 0.74 | 0.51 | 0.79 | -0.10 | -0.39 | 0.44 | -0.40 | -0.22 | -0.50 | -0.39 | -0.39 |
| l | 0.60 | 1.00 | -0.96 | -0.08 | -0.09 | 0.03 | -0.16 | -0.34 | 0.04 | 0.29 | -0.71 | -0.99 | -0.37 | -0.23 |
| v | -0.77 | -0.96 | 1.00 | -0.16 | -0.07 | -0.25 | 0.17 | 0.41 | -0.19 | -0.14 | 0.63 | 0.94 | 0.43 | 0.29 |
| Ab | 0.74 | -0.08 | -0.16 | 1.00 | 0.76 | 0.97 | 0.05 | -0.16 | 0.41 | -0.82 | 0.34 | 0.20 | -0.22 | -0.30 |
| Sp | 0.51 | -0.09 | -0.07 | 0.76 | 1.00 | 0.61 | 0.52 | 0.30 | 0.22 | -0.56 | 0.62 | 0.19 | -0.54 | 0.08 |
| Sk | 0.79 | 0.03 | -0.25 | 0.97 | 0.61 | 1.00 | -0.14 | -0.28 | 0.45 | -0.82 | 0.14 | 0.09 | -0.09 | -0.44 |
| rc | -0.10 | -0.16 | 0.17 | 0.05 | 0.52 | -0.14 | 1.00 | 0.19 | -0.37 | 0.03 | 0.57 | 0.19 | -0.41 | 0.86 |
| Fv/Fm | -0.39 | -0.34 | 0.41 | -0.16 | 0.30 | -0.28 | 0.19 | 1.00 | -0.23 | 0.09 | 0.37 | 0.34 | 0.05 | -0.02 |
| Y | 0.44 | 0.04 | -0.19 | 0.41 | 0.22 | 0.45 | -0.37 | -0.23 | 1.00 | -0.21 | 0.08 | -0.03 | -0.21 | -0.40 |
| ETR | -0.40 | 0.29 | -0.14 | -0.82 | -0.56 | -0.82 | 0.03 | 0.09 | -0.21 | 1.00 | -0.33 | -0.40 | -0.06 | 0.27 |
| E | -0.22 | -0.71 | 0.63 | 0.34 | 0.62 | 0.14 | 0.57 | 0.37 | 0.08 | -0.33 | 1.00 | 0.75 | -0.35 | 0.38 |
| A | -0.50 | -0.99 | 0.94 | 0.20 | 0.19 | 0.09 | 0.19 | 0.34 | -0.03 | -0.40 | 0.75 | 1.00 | 0.34 | 0.19 |
| WUE | -0.39 | -0.37 | 0.43 | -0.22 | -0.54 | -0.09 | -0.41 | 0.05 | -0.21 | -0.06 | -0.35 | 0.34 | 1.00 | -0.17 |
| RWC | -0.39 | -0.23 | 0.29 | -0.30 | 0.08 | -0.44 | 0.86 | -0.02 | -0.40 | 0.27 | 0.38 | 0.19 | -0.17 | 1.00 |
| | drought | | | | | | | | | | | | | |
| R | 1.00 | 0.96 | 0.61 | 0.78 | 0.17 | 0.74 | -0.94 | 0.21 | -0.21 | -0.14 | -0.25 | -0.88 | -0.71 | -0.16 |
| l | 0.96 | 1.00 | 0.64 | 0.89 | 0.12 | 0.86 | -0.82 | 0.45 | -0.18 | 0.10 | -0.50 | -0.84 | -0.51 | 0.11 |
| v | 0.61 | 0.64 | 1.00 | 0.74 | 0.18 | 0.64 | -0.49 | 0.14 | -0.36 | -0.18 | -0.52 | -0.52 | -0.17 | 0.32 |
| Ab | 0.78 | 0.89 | 0.74 | 1.00 | 0.22 | 0.90 | -0.57 | 0.64 | 0.02 | 0.19 | -0.65 | -0.72 | -0.31 | 0.36 |
| Sp | 0.17 | 0.12 | 0.18 | 0.22 | 1.00 | -0.17 | -0.22 | 0.10 | 0.65 | -0.54 | 0.10 | -0.31 | -0.37 | -0.23 |
| Sk | 0.74 | 0.86 | 0.64 | 0.90 | -0.17 | 1.00 | -0.53 | 0.54 | -0.21 | 0.40 | -0.62 | -0.63 | -0.22 | 0.36 |
| rc | -0.94 | -0.82 | -0.49 | -0.57 | -0.22 | -0.53 | 1.00 | 0.07 | 0.25 | 0.33 | -0.03 | 0.85 | 0.87 | 0.44 |
| Fv/Fm | 0.21 | 0.45 | 0.14 | 0.64 | 0.10 | 0.54 | 0.07 | 1.00 | 0.33 | 0.57 | -0.76 | -0.17 | 0.26 | 0.70 |
| Y | -0.21 | -0.18 | -0.36 | 0.02 | 0.65 | -0.21 | 0.25 | 0.33 | 1.00 | -0.08 | 0.18 | 0.03 | -0.06 | -0.11 |
| ETR | -0.14 | 0.10 | -0.18 | 0.19 | -0.54 | 0.40 | 0.33 | 0.57 | -0.08 | 1.00 | -0.60 | 0.04 | 0.52 | 0.61 |
| E | -0.25 | -0.50 | -0.52 | -0.65 | 0.10 | -0.62 | -0.03 | -0.76 | 0.18 | -0.60 | 1.00 | 0.24 | -0.45 | -0.89 |
| A | -0.88 | -0.84 | -0.52 | -0.72 | -0.31 | -0.63 | 0.85 | -0.17 | 0.03 | 0.04 | 0.24 | 1.00 | 0.69 | 0.20 |
| WUE | -0.71 | -0.51 | -0.17 | -0.31 | -0.37 | -0.22 | 0.87 | 0.26 | -0.06 | 0.52 | -0.45 | 0.69 | 1.00 | 0.76 |

| | | | | | | | | | | | | | | |
|---|---|---|---|---|---|---|---|---|---|---|---|---|---|---|
| RWC | -0.16 | 0.11 | 0.32 | 0.36 | -0.23 | 0.36 | 0.44 | 0.70 | -0.11 | 0.61 | -0.89 | 0.20 | 0.76 | 1.00 |
